# 20-year trends in cause-specific heart failure outcomes by sex, socioeconomic status, and place of diagnosis: a population-based study

**DOI:** 10.1016/S2468-2667(19)30108-2

**Published:** 2019-07-31

**Authors:** Claire A Lawson, Francesco Zaccardi, Iain Squire, Suping Ling, Melanie J Davies, Carolyn S P Lam, Mamas A Mamas, Kamlesh Khunti, Umesh T Kadam

**Affiliations:** a Diabetes Research Centre, University of Leicester, Leicester, UK; b NIHR Leicester Biomedical Research Centre, Cardiovascular Research Centre, Glenfield General Hospital, Leicester University, Leicester, UK; c National Heart Centre, Duke-NUS Medical School, Singapore; d University Medical Centre Groningen, Groningen, Netherlands; e The George Institute for Global Health, Newton, NSW, Australia; f Keele Cardiovascular Research Group, Centre for Prognosis Research, Keele University, Staffordshire, UK

## Abstract

**Background:**

Heart failure is an important public health issue affecting about 1 million people in the UK, but contemporary trends in cause-specific outcomes among different population groups are unknown.

**Methods:**

In this retrospective, population-based study, we used the UK Clinical Practice Research Datalink and Hospital Episodes Statistics databases to identify a cohort of patients who had a diagnosis of incident heart failure between Jan 1, 1998, and July 31, 2017. Patients were eligible for inclusion if they were aged 30 years or older with a first code for heart failure in their primary care or hospital record during the study period. We assessed cause-specific admission to hospital (ie, hospitalisation) and mortality, by age, sex, socioeconomic status, and place of diagnosis (ie, hospital *vs* community diagnosis). We calculated outcome rates separately for the first year (first-year rates) and for the second-year onwards (subsequent-year rates). Patients were followed up until death or study end. This study is registered with Clinical Practice Research Datalink Independent Scientific Advisory Committee, protocol number 18_037R.

**Findings:**

We identified 88 416 individuals with incident heart failure over the study period, of whom 43 461 (49%) were female. The mean age was 77·8 years (SD 11·3) and median follow-up was 2·4 years (IQR 0·5 to 5·7). Age-adjusted first-year rates of hospitalisation increased by 28% for all-cause admissions, from 97·1 (95% CI 94·3 to 99·9) to 124·2 (120·9 to 127·5) per 100 person-years; by 28% for heart failure-specific admissions, from 17·2 (16·2 to 18·2) to 22·1 (20·9 to 23·2) per 100 person-years; and by 42% for non-cardiovascular admissions, from 59·2 (57·2 to 61·2) to 83·9 (81·3 to 86·5) per 100 person-years. 167 641 (73%) of 228 113 hospitalisations were for non-cardiovascular causes and annual rate increases were higher for women (3·9%, 95% CI 2·8 to 4·9) than for men (1·4%, 0·6 to 2·1; p<0·0001); and for patients diagnosed with heart failure in hospital (2·4%, 1·4 to 3·3) than those diagnosed in the community (1·2%, 0·3 to 2·2). Annual increases in hospitalisation due to heart failure were 2·6% (1·9 to 3·4) for women compared with stable rates in men (0·6%, −0·9 to 2·1), and 1·6% (0·6 to 2·6) for the most deprived group compared with stable rates for the most affluent group (1·2%, −0·3 to 2·8). A significantly higher risk of all-cause hospitalisation was found for the most deprived than for the most affluent (incident rate ratio 1·34, 95% CI 1·32 to 1·35) and for the hospital-diagnosed group than for the community-diagnosed group (1·76, 1·73 to 1·80). Age-adjusted first-year rates of all-cause mortality decreased by 6% from 24·5 (95% CI 23·4 to 39·2) to 23·0 (22·0 to 24·1) per 100 person-years. Annual change in mortality was −1·4% (95% CI −2·3 to −0·5) in men but was stable for women (0·3%, −0·5 to 1·1), and −2·7% (–3·2 to −2·2) for the community-diagnosed group compared with −1·1% (–1·8 to −0·4) in the hospital-diagnosed group (p<0·0001). A significantly higher risk of all-cause mortality was seen in the most deprived group than in the most affluent group (hazard ratio 1·08, 95% CI 1·05 to 1·11) and in the hospital-diagnosed group than in the community-diagnosed group (1·55, 1·53 to 1·58).

**Interpretation:**

Tailored management strategies and specialist care for patients with heart failure are needed to address persisting and increasing inequalities for men, the most deprived, and for those who are diagnosed with heart failure in hospital, and to address the worrying trends in women.

**Funding:**

Wellcome Trust.

## Introduction

Heart failure is a global pandemic affecting approximately 23 million people worldwide including 6 million people in the USA,^1^ more than 15 million people in Europe, and 1 million in the UK alone.^2^, ^3^ Despite improvements in heart failure therapies over the past two decades, risk of mortality remains high,^4^ with inequalities reported among population groups according to factors such as sex and socioeconomic status.^5^, ^6^, ^7^ The burden of non-elective admission to hospital (ie, hospitalisation) is also high, accounting for over two-thirds of the estimated US$108 billion spent on heart failure globally each year.^8^ Furthermore, the economic costs associated with heart failure are likely to increase exponentially over the next decade due to a projected 46% increase in the prevalence of heart failure by 2030,^9^ alongside increasing costs of health care in an ageing population with multiple morbidities. Consequently, attempts to decrease the substantial social, health, and economic burden of heart failure have become a top public health priority.^10^

**Research in context**

**Evidence before this study**

Heart failure affects approximately 23 million people worldwide and is associated with adverse clinical outcomes (high burden of admissions to hospital, costs, and mortality). On Sept 17, 2018, we searched PubMed for observational studies published in English since Jan 1, 2000, reporting outcome trends in heart failure. We used the term “heart failure” in different combinations with “surviv*”, “rate*”, “outcome*”, “mortality”, “death*”, “hospital*”, “prognos*”, and “trend*”. We reviewed the full text of relevant articles to assess appropriateness for inclusion. Most studies were from the USA and focused on specific care settings (hospital or community) that are unable to ascertain date of incident heart failure accurately, or on single communities, which might not reflect the general population. No studies had reported on cause-specific outcome trends in a national sample of patients with incident heart failure, regardless of place of diagnosis, among different population groups.

**Added value of this study**

By linking national general practice, hospital, and death data, we were able to report outcome burden after newly diagnosed heart failure and make group comparisons. Rates of admission to hospital (ie, hospitalisations) are increasing and, although mortality has improved slightly over the past 20 years, mortality risk is higher than previously reported in non-incident cohorts. Furthermore, we found inequities in outcomes between different heart failure populations, some of which persisted or worsened over time. Risks of all outcomes were higher in the older age groups than in the younger age groups, for men than for women, the most deprived than for the most affluent, and for those first diagnosed with heart failure in hospital than those diagnosed in the community. We also observed worrying trends for women with heart failure, with worsening outcomes over the past two decades. By investigating cause-specific outcomes, we showed that these disparities probably reflect the growing burden of non-cardiovascular comorbidities in patients with heart failure, requiring contemporary prevention and management approaches.

**Implications of all the available evidence**

In people with heart failure, persisting high mortality and increasing hospitalisation rates are a substantial social, health, and economic burden. The increasing number of people first diagnosed with heart failure in hospital, with associated significantly worse outcomes, calls for early recognition and timely specialist intervention. Furthermore, diverging outcome trends over two decades among population groups according to age, sex, socioeconomic status, and place of diagnosis calls for tailored public health prevention approaches to reduce inequalities and improve outcomes for the highest risk groups.

Previous epidemiological evidence in heart failure has predominantly focused on mortality in high-risk cohorts who have been admitted to hospital,^11^, ^12^, ^13^ low-risk primary care cohorts,^14^ or selected communities,^5^, ^6^, ^15^ which might not represent the general population of people who have heart failure and restrict the potential for group comparisons. To develop tailored public health prevention approaches and plan health services effectively, contemporary population-level data on emerging outcome trends among different groups of populations with heart failure are required. Information on cause-specific rates of hospitalisation in people with heart failure is particularly important to meet the projected increase in demands. Through linkage of primary care, hospital, and mortality data, we investigated differences in cause-specific outcomes and trends among groups with incident heart failure according to age group, sex, socioeconomic status, and place of diagnosis.

## Methods

### Study design and participants

We used the Clinical Practice Research Datalink (CPRD) and Hospital Episodes Statistics (HES) databases to identify a cohort of patients in England, UK, with incident heart failure diagnosed between Jan 1, 1998, and July 31, 2017. The CPRD is one of the largest longitudinal, linked, anonymised databases in the world and contains approximately 7% of the UK general population, representative of the population's sex and age. It includes patient demographics, risk factors, symptoms, clinical diagnoses, investigations, referrals, and prescriptions and has been validated for epidemiological research.^16^ HES includes all UK National Health Service (NHS) admissions in England. The Independent Scientific Advisory Committee provided permission for data access (protocol identifier 18_037R).

We included all patients aged 30 years or older with a first code for heart failure in their primary care or hospital record during the 20-year study period. To ascertain the diagnostic date of heart failure accurately, patients were only included if their CPRD and HES records were eligible for linkage (ie, the primary care centre had consented to have their records linked) and they had been registered with a CPRD primary care practice for a minimum of 1 year before heart failure diagnosis. The date of heart failure diagnosis was the date of study entry. We used updated, clinically validated heart failure CPRD codes^17^ and primary International Classification of Diseases-10 (ICD-10) discharge codes (appendix pp 6–7). If patients had codes in both records, the first chronologically was assigned as the date of heart failure diagnosis.

### Procedures

Patients were categorised into exposure groups defined by age (<70 years, 70–79 years, 80–89 years, and ≥90 years), sex, socioeconomic status, and place of diagnosis. Socioeconomic status was based on the 2010 patient-level Index of Multiple Deprivation score,^18^ which was ranked into quintiles (the most affluent group [quintile 1] to the most deprived group [quintile 5]). Place of diagnosis was defined by where the individual had been diagnosed with heart failure, in the community or hospital, on the basis of the first heart-failure code.

Using the most recent value before study entry, we categorised each patient's baseline characteristics associated with heart failure risk including current smoking and alcohol (yes or no), body-mass index (BMI), systolic blood pressure, cholesterol, haemoglobin concentration, and estimated glomerular filtration rate (eGFR), which was calculated using the Chronic Kidney Disease Epidemiology Collaboration formula.^19^ Prescribed drugs, including β blockers, angiotensin-converting enzyme inhibitors, angiotensin II receptor blockers, aldosterone receptor antagonists, aspirin, and loop diuretics, were identified by at least one prescription in a 4-month period before diagnosis of heart failure. Comorbidities were identified by at least one Read Code in CPRD or ICD-10 code in HES at any time up to and including the date of heart failure diagnosis.

### Outcomes

Our outcomes were cause-specific hospitalisations and mortality. Hospitalisations were defined as including all non-elective admissions with at least one overnight stay and occurring after but not including the date of heart failure diagnosis. Overall number of hospitalisations during follow-up after heart failure diagnosis were counted for each patient and further stratified by the following cause-specific admissions: primary heart failure, other cardiovascular disease, and non-cardiovascular disease admissions according to the primary discharge code. Date and cause of death was ascertained from linked Office of National Statistics mortality records. We coded deaths as “cardiovascular” when an ICD-10, chapter 9 code was recorded in the primary position and “non-cardiovascular disease” for all remaining deaths.

For both outcomes follow-up was until death or study end (July 31, 2017).

### Statistical analysis

Descriptive statistics are presented as number and proportion for dichotomous data, mean (SD) for continuous data, and median (IQR) for skewed continuous data. We did trend analyses in the whole group and stratified by age groups, sex, socioeconomic status (most affluent quintile, most deprived quintile), and place of diagnosis (community, hospital). Given the increased risk of poor outcomes in the first year after diagnosis of heart failure, we calculated outcome rates separately for the first year (first-year rates) and for the second year onwards in those who survived their first year (subsequent-year rates). Additionally, due to the high risk of death in the first month after diagnosis of heart failure, we only calculated first-year mortality rates in survivors of the first month. We estimated outcome rates at the mean population age using age-adjusted negative binomial models. We did three trend measurements: percentage difference in outcomes rates (measured per 100 person-years) between two 4-year time periods of heart failure diagnosis (1998–2001 and 2012–15) calculated as 100*([rate in time period 2 – rate in time period 1]/rate in time period 1); average annual percentage change in outcome rates; and change in trends using Joinpoint regression, which is a statistical software that calculates annual percentage change in rate and tests whether any apparent change in trend is significant.^20^ We investigated differences in trends between population groups by fitting an interaction term (group*year of heart failure diagnosis) to the negative binomial models already containing age. Because interaction tests have low power, we also examined trends visually by plotting graphs of outcome rates by year of heart failure diagnosis to aid interpretation.

For overall differences between groups, we compared each older age group with the youngest age group (<70 years; reference), females with males (reference), lower socioeconomic quintiles (quintile 2 to 5) with the most affluent quintile (quintile 1; reference), and the hospital-diagnosed group with the community-diagnosed group (reference). We investigated differences in total and cause-specific hospitalisation rates among groups using negative binomial models to estimate incidence rate ratios (IRR) with 95% CIs, presenting unadjusted and adjusted for all covariates (ie, age, sex, socioeconomic status, ethnicity, place of diagnosis, calendar year of diagnosis, β blocker, angiotensin-converting enzyme inhibitor, angiotensin receptor blocker, aldosterone antagonist, aspirin, loop diuretic, number of comorbidities, ischaemic heart disease, myocardial infarction, atrial fibrillation, hypertension, diabetes, stroke, anaemia, obesity, chronic kidney disease, chronic obstructive pulmonary disease, asthma, depression, osteoarthritis, cancer and dementia, smoking, alcohol, BMI, systolic blood pressure, cholesterol, haemoglobin, and eGFR). For time-to-mortality outcomes, we used Royston-Parmer flexible parametric survival models to do age-standardised survival predictions at 1, 3, and 5 years after diagnosis of heart failure for the whole group and population subgroups by the earliest and latest 4-year time period of diagnosis (ie, 1998–2001 and 2012–15). Next, we plotted age-standardised and calendar-year-standardised survival curves (all-cause mortality) and cumulative incidence curves (cause-specific mortality).^21^ Finally, we estimated hazard ratios (HRs), unadjusted and adjusted for all covariates, with 95% CIs, comparing each subgroup with their respective reference group for all-cause and cause-specific death.

To account for missing data in the multivariable models (p 8), we did multiple imputations using matching variables and full-conditional specification, using the MI Impute command, and results were obtained using Rubin's rules.^22^ We did sensitivity analyses to assess complete-case analysis, in which we removed patients for whom missing values had been imputed, and stratification of adjusted outcome effects by calendar year of diagnosis.

We did all analyses using Stata-MP 14.

### Role of the funding source

The funder of the study had no role in study design, data collection, data analysis, data interpretation, or writing of the report. The corresponding author had full access to all the data and had final responsibility for the decision to submit for publication

## Results

We identified 88 416 patients with incident heart failure over the 20-year study period, of whom 43 461 (49%) were female, with a mean age of 77·8 years (SD 11·3; table 1), and a median follow-up time of 2·4 years (IQR 0·5–5·7; appendix p 2). Of the study population, 36 415 (41%) patients were first diagnosed with heart failure in hospital; a proportion that increased exponentially over time from 26% in 1998 to 74% in 2017 (appendix p 3).

**Table 1:** Patient characteristics by sex, socio-economic status, and place of diagnosis

			**All (n=88 416)**	**Sex**		**Socioeconomic status**					**Place of diagnosis**	
				Male (n=44 955)	Female (n=43 461)	Quintile 1 (most affluent; n=16 481)	Quintile 2 (n=20 468)	Quintile 3 (n=18 910)	Quintile 4 (n=17 542)	Quintile 5 (most deprived; n=14 861)	Community (n=52 001)	Hospital (n=36 415)
**Demographic**												
Age, years			77·8 (11·3)	75·3 (11·5)	80·3 (10·5)	79·0 (10·8)	78·7 (10·8)	78·2 (11·2)	76·9 (11·5)	75·6 (12·0)	76·9 (11·2)	79·0 (11·4)
	<70		18 168 (21%)	12 178 (27%)	5990 (14%)	2884 (17%)	3659 (18%)	3664 (19%)	3984 (23%)	3953 (27%)	11 627 (22%)	6541 (18%)
	70–79		25 878 (29%)	14 379 (32%)	11 499 (26%)	4638 (28%)	5804 (28%)	5416 (29%)	5336 (30%)	4653 (31%)	16 357 (31%)	9521 (26%)
	80–89		33 152 (37%)	14 956 (33%)	18 196 (42%)	6542 (40%)	8104 (40%)	7310 (39%)	6287 (36%)	4837 (33%)	18 697 (36%)	14 455 (40%)
	≥90		11 218 (13%)	3442 (8%)	7776 (18%)	2417 (15%)	2901 (14%)	2520 (13%)	1935 (11%)	1418 (10%)	5320 (10%)	5898 (16%)
**Sex**												
	Female		43 461 (49%)	N/A	N/A	7804 (47%)	9889 (48%)	9258 (49%)	8879 (51%)	7552 (51%)	24 700 (47%)	18 761 (52%)
	Male		44 461 (51%)	N/A	N/A	8677 (53%)	10 579 (52%)	9652 (51%)	8663 (49%)	7309 (49%)	27 301 (53%)	17 654 (48%)
**Socioeconomic group**												
	Most affluent group		16 481 (19%)	8677 (19%)	7804 (18%)	N/A	N/A	N/A	N/A	N/A	10 011 (19%)	6470 (18%)
	Most deprived group		14 861 (17%)	7309 (16%)	7552 (17%)	N/A	N/A	N/A	N/A	N/A	8463 (16%)	6398 (18%)
**Place of diagnosis**												
	Hospital		36 415 (41%)	17 654 (39%)	18 761 (43%)	6470 (39%)	8384 (41%)	7662 (41%)	7441 (42%)	6398 (43%)	N/A	N/A
	Community		52 001 (59%)	27 301 (61%)	24 700 (57%)	10 011 (61%)	12 084 (59%)	11 248 (59%)	10 101 (58%)	8463 (57%)	N/A	N/A
**Clinical**												
**Medication**												
	β blocker		26 627 (30%)	14 222 (32%)	12 405 (29%)	5322 (32%)	6225 (30%)	5619 (30%)	5224 (30%)	4209 (28%)	17 134 (33%)	9493 (26%)
	Angiotensin-converting enzyme inhibitor		33 083 (37%)	18 350 (41%)	14 733 (34%)	6040 (37%)	7717 (38%)	7054 (37%)	6633 (38%)	5596 (38%)	21 978 (42%)	11 105 (31%)
	Angiotensin receptor blocker		9166 (10%)	4354 (10%)	4812 (11%)	2009 (12%)	2171 (11%)	1905 (10%)	1736 (10%)	1332 (9%)	5541 (11%)	3625 (10%)
	Angiotensin-converting enzyme inhibitor or angiotensin receptor blocker		40 983 (46%)	22 031 (49%)	18 952 (44%)	7795 (47%)	9567 (47%)	8715 (46%)	8129 (46%)	6723 (45%)	26 689 (51%)	14 294 (39%)
	Aldosterone antagonist*		5767 (7%)	3108 (7%)	2659 (6%)	1154 (7%)	1348 (7%)	1200 (6%)	1148 (7%)	908 (6%)	3432 (7%)	2335 (6%)
	Aspirin		33 908 (38%)	18 697 (42%)	15 211 (35%)	6068 (37%)	7719 (38%)	7303 (39%)	6855 (39%)	5911 (40%)	21 404 (41%)	12 504 (34%)
	Diuretic (loop)		43 332 (49%)	21 412 (48%)	21 920 (50%)	7987 (48%)	10 113 (49%)	9274 (49%)	8568 (49%)	7307 (49%)	28 139 (54%)	15 193 (42%)
**Comorbidities**												
	Mean number		4·1 (2·0)	4·0 (2·0)	4·2 (2·1)	3·9 (2·0)	4·0 (2·0)	4·1 (2·0)	4·2 (2·1)	4·2 (2·1)	3·7 (2·0)	4·6 (2·1)
		<2	8634 (10%)	4760 (11%)	3874 (9%)	1730 (10%)	2068 (10%)	1860 (10%)	1601 (9%)	1343 (9%)	6386 (12%)	2248 (6%)
		2–3	27 676 (31%)	14 798 (33%)	12 878 (30%)	5416 (33%)	6476 (32%)	6041 (32%)	5307 (30%)	4387 (30%)	18 495 (36%)	9181 (25%)
		4–5	31 205 (35%)	15 785 (35%)	15 420 (35%)	5834 (35%)	7396 (36%)	6566 (35%)	6193 (35%)	5171 (35%)	17 699 (34%)	13 506 (37%)
		>5	20 901 (24%)	9612 (21%)	11 289 (26%)	3501 (21%)	4528 (22%)	4443 (23%)	4441 (25%)	3960 (27%)	9421 (18%)	11 480 (32%)
Ischaemic heart disease			43 983 (50%)	25 111 (56%)	18 872 (43%)	7979 (48%)	9891 (48%)	9356 (49%)	8919 (51%)	7779 (52%)	24 165 (46%)	19 818 (54%)
Myocardial infarction			23 481 (27%)	14 795 (33%)	8686 (20%)	4311 (26%)	5266 (26%)	4984 (26%)	4734 (27%)	4162 (28%)	13 197 (25%)	10 284 (28%)
Atrial fibrillation			34 884 (39%)	17 909 (40%)	16 975 (39%)	6795 (41%)	8361 (41%)	7481 (40%)	6715 (38%)	5483 (37%)	17 332 (33%)	17 552 (48%)
Hypertension			56 908 (64%)	27 768 (62%)	29 140 (67%)	10 603 (64%)	13 135 (64%)	12 068 (64%)	11 416 (65%)	9614 (65%)	30 915 (59%)	25 993 (71%)
Diabetes			21 565 (24%)	11 872 (26%)	9693 (22%)	3439 (21%)	4745 (23%)	4462 (24%)	4688 (27%)	4202 (28%)	10 675 (21%)	10 890 (30%)
Stroke			10 686 (12%)	5556 (12%)	5130 (12%)	1911 (12%)	2386 (12%)	2316 (12%)	2178 (12%)	1878 (13%)	5668 (11%)	5018 (14%)
Anaemia			11 167 (13%)	4419 (10%)	6748 (16%)	1942 (12%)	2488 (12%)	2415 (13%)	2272 (13%)	2027 (14%)	5318 (10%)	5849 (16%)
Obesity			22 207 (25%)	11 319 (25%)	10 888 (25%)	3457 (21%)	4725 (23%)	4706 (25%)	4910 (28%)	4380 (29%)	12 475 (24%)	9732 (27%)
Chronic kidney disease			37 850 (56%)	17 244 (49%)	20 606 (63%)	7361 (56%)	9062 (57%)	8198 (56%)	7349 (56%)	5821 (54%)	20 021 (53%)	17 829 (60%)
Chronic obstructive pulmonary disease			16 438 (19%)	9229 (21%)	7209 (17%)	2282 (14%)	3299 (16%)	3276 (17%)	3688 (21%)	3859 (26%)	8900 (17%)	7538 (21%)
Asthma			16 512 (19%)	7911 (18%)	8601 (20%)	2726 (17%)	3631 (18%)	3371 (18%)	3450 (20%)	3313 (22%)	9488 (18%)	7024 (19%)
Depression			20 060 (23%)	7997 (18%)	12 063 (28%)	3392 (21%)	4310 (21%)	4301 (23%)	4273 (24%)	3754 (25%)	11 461 (22%)	8599 (24%)
Osteoarthritis			32 580 (37%)	13 753 (31%)	18 827 (43%)	6097 (37%)	7612 (37%)	6985 (37%)	6333 (36%)	5490 (37%)	18 462 (36%)	14 118 (39%)
Cancer			20 500 (23%)	10 733 (24%)	9767 (22%)	4227 (26%)	5121 (25%)	4444 (24%)	3773 (22%)	2900 (20%)	11 360 (22%)	9140 (25%)
Dementia			3993 (5%)	1502 (3%)	2491 (6%)	728 (4%)	935 (5%)	896 (5%)	790 (5%)	633 (4%)	1927 (4%)	2066 (6%)
**Current smoking**												
	Yes		17 408 (21%)	9967 (23%)	7441 (18%)	2553 (16%)	3619 (19%)	3554 (20%)	3838 (23%)	3811 (27%)	11 101 (23%)	6307 (18%)
	No		65 786 (79%)	32 942 (77%)	32 844 (82%)	13 040 (84%)	15 659 (81%)	14 179 (80%)	12 677 (77%)	10 132 (73%)	37 499 (77%)	28 287 (82%)
**Alcohol**												
	Yes		53 789 (71%)	31 526 (79%)	22 263 (61%)	10 890 (77%)	12 894 (74%)	11 503 (71%)	10 175 (67%)	8245 (64%)	32 266 (73%)	21 523 (68%)
	No		22 194 (29%)	8172 (21%)	14 022 (39%)	3253 (23%)	4540 (26%)	4675 (29%)	5016 (33%)	4674 (36%)	12 105 (27%)	10 179 (32%)
BMI, kg/m^2^			26·8(23·6–30·8)	27·0(24·1–30·6)	26·6(22·9–31·2)	26·3(23·4–29·8)	26·6(23·5–30·4)	26·8(23·6–30·8)	27·2(23·8–31·4)	27·4(23·8–31·8)	26·8(23·6–30·6)	26·9(23·6–31·2)
Systolic blood pressure, mm Hg			137·9 (21·7)	135·7 (20·9)	140·2 (22·2)	137·3 (21·2)	138·2 (21·7)	138·0 (22·0)	137·8 (21·8)	138·2 (21·6)	138·8 (21·8)	136·7 (21·5)
Cholesterol, mmol/L			4·5(3·8–5·4)	4·3(3·6–5·1)	4·8(4·1–5·7)	4·5(3·8–5·4)	4·5(3·8–5·4)	4·6(3·8–5·4)	4·5(3·8–5·4)	4·5(3·7–5·3)	4·6(3·9–5·5)	4·4(3·7–5·3)
Haemoglobin, g/dL			13·0 (1·9)	13·4 (2·0)	12·5 (1·7)	13·0 (1·9)	13·0 (1·9)	12·9 (1·9)	12·9 (1·9)	13·0 (1·9)	13·1 (1·8)	12·7 (1·9)
eGFR mL/min per m^2^			57·5 (20·9)	60·3 (21·4)	54·5 (19·9)	57·2 (20·1)	56·9 (20·4)	57·2 (20·6)	57·8 (21·2)	59·0 (22·4)	59·2 (20·3)	55·4 (21·4)

Data are mean (SD), n (%), or median (IQR). N/A=not applicable. BMI=body-mass index. eGFR=estimated glomerular filtration rate.

* Spironolactone or eplerenone.

In the first year after diagnosis of heart failure, 35 171 (40%) patients had at least one and 15 787 (20%) had multiple hospitalisations, resulting in a total of 66 664 admissions during the first year after heart failure diagnosis. First-year rates of hospitalisation increased by 28% from 97·1 (95% CI 94·3–99·9) per 100 person-years during 1998–2001 to 124·2 (120·9–127·5) per 100 person-years during 2012–15, but rates began to plateau after 2005 (table 2). The steepest increase in first-year rates of hospitalisation was among people aged 80 years and older with rates for the younger two age groups remaining stable. Subsequent-year rates of hospitalisation were lower than first-year rates and remained stable overall, but with predicted rates decreasing over time for the youngest age group and increasing over time for the oldest age groups (appendix pp 9–11).

**Table 2:** Predicted rates of hospitalisation during the first year after heart failure diagnosis, by population group and calendar year

		**Predicted first-year rate, per 100 person-years**		Relative difference*	**p_interaction_**†	**Average annual percentage change**‡	**Slope change; annual percentage change before and after trend change**§		
		1998–2001	2012–15				Before	Break	After
**All admissions (n=66 664)**									
All		97·1 (94·3 to 99·9)	124·2 (120·9 to 127·5)	28%	··	1·8% (1·2 to 2·4)	3·7% (2·4 to 5·2)	2005	0·4% (–0·2 to 1·1)
Age, years									
	<70	94·1 (88·2 to 100·0)	89·0 (83·9 to 94·0)	–5%	1 (ref)	–0·2% (–1·2 to 0·8)	–0·2% (–1·2 to 0·8)	N/A	–0·2% (–1·2 to 0·8)
	70–79	91·9 (87·4 to 96·3)	107·8 (102·3 to 113·3)	17%	<0·0001	0·9% (–0·1 to 2·0)	3·1% (0·8 to 5·4)	2005	–0·6% (–1·7 to 0·6)
	80–89	98·4 (93·6 to 103·2)	144·6 (138·3 to 150·8)	47%	<0·0001	3·0% (2·4 to 3·7)	6·3% (4·5 to 8·1)	2004	1·3% (0·8 to 1·8)
	≥90	102·0 (92·1 to 111·9)	174·1 (161·6 to 186·7)	71%	<0·0001	4·4% (2·7 to 6·2)	10·9% (5·8 to 16·3)	2004	1·1% (–0·2 to 2·3)
Sex									
	Men	109·9 (105·4 to 114·4)	119·1 (114·8 to 123·5)	8%	1 (ref)	0·5% (–0·1 to 1·0)	0·5% (–0·1 to 1·0)	N/A	0·5% (–0·1 to 1·0)
	Women	85·7 (82·3 to 89·2)	129·7 (124·7 to 134·7)	51%	<0·0001	3·0% (2·1 to 3·9)	6·0% (4·3 to 7·7)	2006	0·4% (–0·7 to 1·5)
Socioeconomic status									
	Most affluent	85·9 (79·8 to 92·1)	109·7 (103·1 to 116·3)	28%	1 (ref)	1·5% (0·0 to 3·1)	4·0% (0·0 to 8·2)	2004	0·2% (–1·1 to 1·5)
	Most deprived	113·2 (105·8 to 120·6)	145·3 (135·6 to 155·1)	28%	0·103	1·9% (0·9 to 2·9)	1·9% (0·9 to 2·9)	N/A	1·9% (0·9 to 2·9)
Place of diagnosis									
	Community	80·0 (77·3 to 82·7)	88·3 (84·9 to 91·6)	10%	1 (ref)	0·7% (–0·2 to 1·6)	4·6% (2·2 to 6·9)	2004	–1·3% (–2·1 to −0·5)
	Hospital	145·0 (137·6 to 152·5)	163·2 (157·3 to 169·0)	13%	0·058	0·9% (0·3 to 1·5)	0·9% (0·3 to 1·5)	N/A	0·9% (0·3 to 1·5)
**Heart failure admissions (n=11 543)**									
All		17·2 (16·2 to 18·2)	22·1 (20·9 to 23·2)	28%	··	1·8% (0·7 to 2·9)	0·8% (–0·1 to 1·7)	2011	5·2% (0·7 to 9·9)
Age, years									
	<70	14·5 (12·6 to 16·3)	14·1 (12·5 to 15·7)	–3%	1 (ref)	–0·5% (–2·7 to 1·9)	–6·5% (–10·1 to −2·8)	2015	5·3% (1·7 to 8·9)
	70–79	16·4 (14·9 to 17·9)	18·1 (16·3 to 20·0)	10%	0·761	0·1% (–0·9 to 1·0)	0·1% (–0·9 to 1·0)	N/A	0·1% (–0·9 to 1·0)
	80–89	18·8 (17·1 to 20·6)	26·9 (24·7 to 29·1)	43%	<0·0001	2·4% (1·7 to 3·2)	2·4% (1·7 to 3·2)	N/A	2·4% (1·7 to 3·2)
	≥90	17·8 (14·4 to 21·2)	35·2 (30·4 to 39·9)	98%	<0·0001	3·7% (1·4 to 6·0)	3·7% (1·4 to 6·0)	N/A	3·7% (1·4 to 6·0)
Sex									
	Men	20·3 (18·7 to 21·9)	22·4 (20·8 to 24·1)	10%	1 (ref)	0·6% (–0·9 to 2·1)	–1·9% (–3·4 to −0·4)	2009	5·3% (1·5 to 9·3)
	Women	14·6 (13·4 to 15·8)	21·6 (20·0 to 23·3)	48%	<0·0001	2·6% (1·9 to 3·4)	2·6% (1·9 to 3·4)	N/A	2·6% (1·9 to 3·4)
Socioeconomic status									
	Most affluent	15·1 (13·0 to 17·3)	19·1 (16·9 to 21·3)	26%	1 (ref)	1·2% (–0·3 to 2·8)	1·2% (–0·3 to 2·8)	N/A	1·2% (–0·3 to 2·8)
	Most deprived	20·5 (18·0 to 23·1)	26·2 (22·9 to 29·5)	28%	0·597	1·6% (0·6 to 2·6)	1·6% (0·6 to 2·6)	N/A	1·6% (0·6 to 2·6)
Place of diagnosis									
	Community	11·3 (10·5 to 12·1)	12·3 (11·3 to 13·2)	9%	1 (ref)	0·2% (–0·9 to 1·3)	0·2% (–0·9 to 1·3)	N/A	0·2% (–0·9 to 1·3)
	Hospital	35·5 (32·2 to 38·7)	32·9 (30·8 to 35·1)	–7%	0·403	–1·6% (–3·7 to 0·6)	–8·5% (–14·4 to −2·1)	2003	1·4% (–0·5 to 3·4)
**Other cardiovascular disease admissions (n=14 776)**									
All		20·3 (19·3 to 21·3)	22·5 (21·4 to 23·5)	11%	··	0·7% (–0·4 to 1·8)	5·3% (2·9 to 7·9)	2005	–2·4% (–3·7 to −1·1)
Age, years									
	<70	23·0 (20·2 to 25·9)	17·8 (15·6 to 20·1)	–23%	1 (ref)	–1·7% (–2·6 to −0·7)	–1·7% (–2·6 to −0·7)	N/A	–1·7% (–2·6 to −0·7)
	70–79	19·9 (18·3 to 21·5)	21·7 (19·8 to 23·6)	9%	0·003	0% (–2·1 to 2·2)	4·5% (–0·2 to 9·3)	2005	–3·0% (–5·5 to −0·4)
	80–89	19·0 (17·3 to 20·8)	24·7 (22·6 to 26·8)	30%	<0·0001	2·4% (1·2 to 3·7)	10·3% (6·7 to 14·1)	2004	–1·6% (–2·6 to 0·6)
	≥90	17·1 (13·6 to 20·6)	27·9 (23·7 to 32·2)	63%	<0·0001	3·3% (–0·1 to 6·8)	9·5% (3·7 to 15·7)	2007	–3·4% (–7·9 to 1·4)
Sex									
	Men	22·6 (21·1 to 24·2)	21·9 (20·5 to 23·3)	–3%	1 (ref)	–0·1% (–1·8 to 1·6)	5·5% (1·1 to 10·1)	2004	–3·0% (–4·6 to −1·4)
	Women	18·2 (16·9 to 19·4)	23·1 (21·6 to 24·7)	27%	<0·0001	1·8% (0·4 to 3·2)	6·7% (3·6 to 9·9)	2005	–1·5% (–3·0 to −0·0)
Socioeconomic status									
	Most affluent	18·6 (16·4 to 20·9)	21·2 (19·0 to 23·4)	14%	1 (ref)	0·8% (–0·8 to 2·5)	7·2% (2·6 to 12·0)	2004	–2·5% (–4·0 to −0·9)
	Most deprived	23·0 (20·5 to 25·5)	23·6 (20·8 to 26·3)	3%	0·942	–0·4% (–2·8 to 2·1)	2·9% (0·2 to 5·7)	2008	–4·9% (–9·9 to 0·3)
Place of diagnosis									
	Community	17·2 (16·2 to 18·3)	18·4 (17·2 to 19·7)	7%	1 (ref)	0·5% (–0·9 to 1·9)	5·9% (2·9 to 9·0)	2005	–3·2 (–4·7 to −1·5)
	Hospital	29·3 (26·7 to 31·9)	27·1 (25·4 to 28·8)	–8%	0·003	–0·5% (–1·7 to 0·8)	3·2% (–0·0 to 6·6)	2004	–2·5% (–3·7 to −1·2)
**Non-cardiovascular disease admissions (n=43 965)**									
All		59·2 (57·2 to 61·2)	83·9 (81·3 to 86·5)	42%	··	2·6% (1·9 to 3·3)	4·2% (2·8 to 5·6)	2006	1·2% (0·3 to 2·1)
Age, years									
	<70	57·1 (51·8 to 62·5)	60·5 (55·0 to 65·9)	6%	1 (ref)	0·6% (–0·9 to 2·2)	0·6% (–0·9 to 2·2)	N/A	0·6% (–0·9 to 2·2)
	70–79	55·7 (52·5 to 58·9)	72·1 (67·8 to 76·5)	29%	0·006	1·9% (0·9 to 2·8)	3·7% (1·6 to 5·9)	2005	0·6% (–0·4 to 1·6)
	80–89	60·0 (56·3 to 63·6)	98·7 (93·4 to 104·0)	65%	<0·0001	3·4% (2·8 to 4·0)	3·4% (2·8 to 4·0)	N/A	3·4% (2·8 to 4·0)
	≥90	66·7 (58·5 to 74·8)	114·7 (103·6 to 125·7)	72%	<0·0001	4·2% (2·8 to 5·6)	8·3% (4·9 to 11·8)	2005	1·4% (0·1 to 2·8)
Sex									
	Men	66·3 (63·1 to 69·6)	79·4 (76·1 to 82·8)	20%	1 (ref)	1·4% (0·6 to 2·1)	1·4% (0·6 to 2·1)	N/A	1·4% (0·6 to 2·1)
	Women	52·9 (50·4 to 55·5)	88·9 (84·9 to 92·8)	68%	<0·0001	3·9% (2·8 to 4·9)	7·1% (5·1 to 9·3)	2006	1·0% (–0·2 to 2·3)
Socioeconomic status									
	Most affluent	50·9 (46·6 to 55·2)	73·4 (68·3 to 78·6)	44%	1 (ref)	2·2% (1·3 to 3·1)	2·2% (1·3 to 3·1)	N/A	2·2% (1·3 to 3·1)
	Most deprived	70·0 (64·7 to 75·4)	101·7 (93·9 to 109·5)	45%	0·167	2·8% (1·4 to 4·3)	2·8% (1·4 to 4·3)	N/A	2·8% (1·4 to 4·3)
Place of diagnosis									
	Community	51·6 (49·5 to 53·6)	60·8 (58·1 to 63·5)	18%	1 (ref)	1·2% (0·3 to 2·2)	4·2% (1·6 to 6·8)	2004	–0·4% (–1·2 to 0·5)
	Hospital	80·4 (75·4 to 85·3)	109·1 (104·5 to 113·6)	36%	<0·0001	2·4% (1·4 to 3·30)	2·4% (1·4 to 3·30)	N/A	2·4% (1·4 to 3·30)

Data in parentheses are 95% CIs. With the exception of age groups, all predictions are estimated at the mean population age (77·8 years). Follow-up was until death or study end. N/A=not applicable.

* Relative percentage difference in admission rates (per 100 person-years) between the first and second diagnosis time periods.

† p value for the difference in interaction between calendar year and group.

‡ Average annual percentage change in rates (per 100 person-years) for each increasing year of diagnosis.

§ Change in slope estimated using Joinpoint regression.

**Figure 1:**
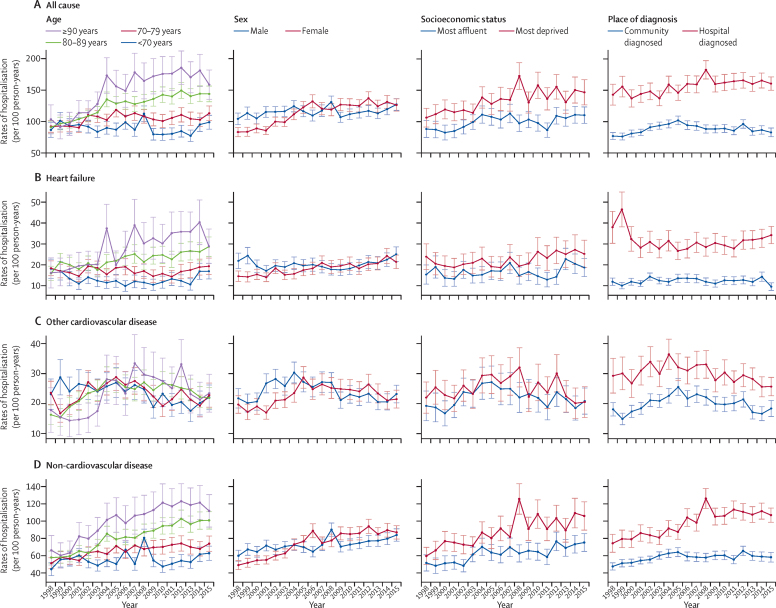
Predicted rates of cause-specific hospitalisation during the first year after heart failure diagnosis, by population group Data are predicted hospitalisation rates, estimated at the mean population age (77·8 years), per 100 person-years by cause, between 1998 and 2015. Whiskers are 95% CIs. Hospitalisations included all non-elective admissions with at least one overnight stay and occurring after but not including the date of heart failure diagnosis. Follow-up was until death or study end.

Heart failure was listed as the primary cause for 11 543 (17%) of 66 664 hospitalisations occurring in the first year after heart failure diagnosis, and 16 623 (10%) of 161 449 hospitalisations in subsequent years. First-year rates of admission for heart failure increased from 17·2 (95% CI 16·2–18·2) per 100 person-years to 22·1 (20·9–23·2) per 100 person-years, at an average increase of 1·8% per annum (table 2), whereas subsequent-year rates remained stable (appendix pp 9–11). This steady increase was in contrast with first-year hospitalisations due to other cardiovascular disease, which decreased by 2·4% per annum after 2005 (table 2). Patterns in first-year rates of hospitalisation by cause of admission, population group, and year are shown in figure 1. Of 228 113 total hospitalisations, non- cardiovascular events were the predominant cause (n=167 641 [73%]), with first-year rates increasing from 59·2 (95% CI 57·2–61·2) per 100 person-years to 83·9 (81·3–86·5) per 100 person-years, an average increase of 2·6% per annum (table 2). Subsequent-year rates were also high and increased by 0·8% per annum, reaching 64·0 (95% CI 62·3–65·8) per 100 person-years during 2012–15 (appendix pp 9–11).

**Figure 2:**
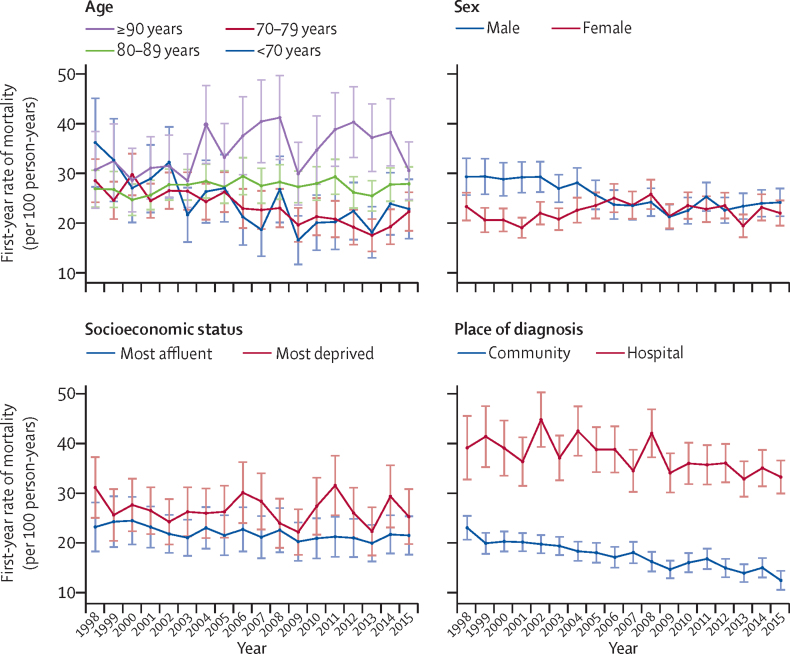
First-year rates of mortality, by population group and calendar year of diagnosis Data are predicted rates of mortality per 100 person-years at mean population age (77·8 years) per 100 person-years between 1998 and 2015. Whiskers are 95% CIs. Rates were calculated in survivors of the first month after heart failure diagnosis.

During follow-up, 65 063 (74%) patients died, with 32 623 (50%) due to cardiovascular events and 32 440 (50%) due to non-cardiovascular disease events. During 1998–2001, 2397 (14%) of 17 550 patients died during the first month after heart failure diagnosis, whereas during 2012–15 this number decreased to 1967 (11%) of 17 834 patients (table 3). In those who survived the first month after their diagnosis of heart failure, age-adjusted first-year mortality rates decreased slightly from 24·5 (95% CI 23·4–25·7) per 100 person-years to 23·0 (22·0–24·1) per 100 person-years, but differences were seen by age (table 3). The youngest two age groups had decreases in mortality whereas the oldest groups had a significant increase in mortality. In the group aged 90 years and older, mortality increased by 20%, reaching 69·6 (95% CI 63·2–76·0) per 100 person-years in 2012–15 (table 3). First-year rates of mortality by calendar year and population group are shown in figure 2. Subsequent-year rates of mortality were lower than first-year rates and decreased by 16% over time, reaching 14·7 (95% CI 14·3–15·2) per 100 person-years in 2012–15 (table 3). Between 1998–2001 and 2012–15, age-standardised mortality risk after heart failure diagnosis decreased from 32% to 29% at 1 year, from 50% to 46% at 3 years, and from 63% to 59% at 5 years (appendix p 12).

**Table 3:** Predicted mortality after heart failure diagnosis, by population group and calendar year

		**First month all-cause deaths**		**Predicted first-year mortality, per 100 person-years**		Relative difference*	**p_interaction_**†	**Average annual percent change**‡	**Slope change; annual percent change before and after break**§		
		1998–2001 (n=17 550)	2012–15 (n=17 834)	1998–2001	2012–15				Before	Break	After
**First-year rates**¶											
All		2397 (14%)	1967 (11%)	24·5 (23·4 to 25·7)	23·0 (22·0 to 24·1)	−6%	..	−0·5% (−0·9 to −0·2)	−0·5% (−0·9 to −0·2)	N/A	−0·5% (−0·9 to −0·2)
Age, years											
	<70	328 (10%)	186 (5%)	13·6 (12·1 to 15·1)	8·9 (7·9 to 10·0)	−35%	1 (ref)	−3·6% (−4·9 to −2·3)	−3·6% (−4·9 to −2·3)	N/A	−3·6% (−4·9 to −2·3)
	70–79	684 (11%)	301 (7%)	21·8 (20·2 to 23·4)	15·8 (14·4 to 17·3)	−28%	0·113	−2·3% (−3·0 to −1·6)	−2·3% (−3·0 to −1·6)	N/A	−2·3% (−3·0 to −1·6)
	80–89	1004 (16%)	901 (13%)	32·6 (30·4 to 34·8)	34·1 (31·9 to 36·3)	5%	<0·0001	0·3% (−0·1 to 0·8)	0·3% (−0·1 to 0·8)	N/A	0·3% (−0·1 to 0·8)
	≥90	381 (21%)	579 (22%)	58·1 (51·5 to 64·7)	69·6 (63·2 to 76·0)	20%	<0·0001	1·2% (0·0 to 2·4)	1·2% (0·0 to 2·4)	N/A	1·2% (0·0 to 2·4)
Sex											
	Men	1103 (13%)	926 (10%)	29·2 (27·4 to 31·0)	23·5 (22·1 to 24·9)	−20%	1 (ref)	−1·4% (−2·3 to −0·5)	−2·6% (−3·4 to −1·7)	2009	0·8% (−1·7 to 3·3)
	Women	1294 (14%)	1041 (12%)	20·7 (19·5 to 22·0)	22·0 (20·7 to 23·4)	6%	<0·0001	0·3% (−0·5 to 1·1)	0·3% (−0·5 to 1·1)	N/A	0·3% (−0·5 to 1·1)
Socioeconomic status											
	Most affluent	375 (13%)	367 (10%)	23·8 (21·3 to 26·2)	21·1 (19·1 to 23·1)	−11%	1 (ref)	−0·8% (−1·2 to −0·4)	−0·8% (−1·2 to −0·4)	N/A	−0·8% (−1·2 to −0·4)
	Most deprived	476 (14%)	310 (11%)	27·6 (24·8 to 30·4)	25·7 (22·9 to 28·5)	−7%	0·46	−0·2% (−1·2 to 0·8)	−0·2% (−1·2 to 0·8)	N/A	−0·2% (−1·2 to 0·8)
Place of diagnosis											
	Community	1449 (11%)	606 (7%)	20·8 (19·7 to 21·8)	14·2 (13·2 to 15·1)	−32%	1 (ref)	−2·7% (−3·2 to −2·2)	−2·7% (−3·2 to −2·2)	N/A	−2·7% (−3·2 to −2·2)
	Hospital	948 (19%)	1361 (15%)	38·8 (35·8 to 41·8)	34·3 (32·3 to 36·2)	−12%	<0·0001	−1·1% (−1·8 to −0·4)	−1·1% (−1·8 to −0·4)	N/A	−1·1% (−1·8 to −0·4)
**Subsequent-year rates**‖											
All		5475 (31%)	5143 (29%)	17·6 (17·3 to 18·0)	14·7 (14·3 to 15·2)	−16%	..	−1·3% (−1·6 to −1·1)	−1·3% (−1·6 to −1·1)	N/A	−1·3% (−1·6 to −1·1)
Age, years											
	<70	682 (20%)	479 (13%)	8·0 (7·7 to 8·4)	5·4 (4·9 to 5·9)	−33%	1 (ref)	−2·9% (−3·6 to −2·1)	−2·9% (−3·6 to −2·1)	N/A	−2·9% (−3·6 to −2·1)
	70–79	1638 (27%)	872 (19%)	14·6 (14·1 to 15·0)	11·3 (10·5 to 12·0)	−23%	0·001	−1·9% (−2·2 to −1·6)	−1·9% (−2·2 to −1·6)	N/A	−1·9% (−2·2 to −1·6)
	80–89	2274 (36%)	2363 (35%)	23·7 (23·0 to 24·5)	20·8 (19·8 to 21·7)	−12%	<0·0001	−0·9% (−1·3 to −0·6)	−0·9% (−1·3 to −0·6)	N/A	−0·9% (−1·3 to −0·6)
	≥90	881 (49%)	1429 (54%)	38·3 (35·8 to 40·8)	41·7 (38·8 to 44·7)	9%	<0·0001	0·4% (−0·1 to 1·0)	0·4% (−0·1 to 1·0	N/A	0·4% (−0·1 to 1·0
Sex											
	Men	2630 (31%)	2501 (27%)	20·2 (19·6 to 20·7)	15·1 (14·4 to 15·8)	−25%	1 (ref)	−2·2% (−2·5 to −1·9)	−2·2% (−2·5 to −1·9)	N/A	−2·2% (−2·5 to −1·9)
	Women	2845 (31%)	2642 (31%)	15·8 (15·4 to 16·2)	14·2 (13·5 to 14·8)	−10%	<0·0001	−0·8% (−1·1 to −0·5)	−0·8% (−1·1 to −0·5)	N/A	−0·8% (−1·1 to −0·5)
Socioeconomic status											
	Most affluent	908 (31%)	1000 (28%)	16·2 (15·5 to 16·9)	13·1 (12·2 to 14·0)	−19%	1 (ref)	−1·4% (−2·1 to −0·7)	−1·4% (−2·1 to −0·7)	N/A	−1·4% (−2·1 to −0·7)
	Most deprived	1056 (32%)	782 (29%)	20·2 (19·4 to 21·1)	16·9 (15·5 to 18·2)	−16%	0·60	−1·3% (−1·8 to −0·9)	−1·3% (−1·8 to −0·9)	N/A	−1·3% (−1·8 to −0·9)
Place of diagnosis											
	Community	3452 (27%)	1656 (19%)	16·7 (16·3 to 17·1)	12·0 (11·5 to 12·5)	−28%	1 (ref)	−2·4% (−2·7 to −2·1)	−2·4% (−2·7 to −2·1)	N/A	−2·4% (−2·7 to −2·1)
	Hospital	2023 (31%)	3487 (38%)	21·4 (20·6 to 22·2)	18·7 (17·9 to 19·5)	−13%	<0·0001	−1·0% (−1·3 to −0·7)	−1·0% (−1·3 to −0·7)	N/A	−1·0% (−1·3 to −0·7)

Crude mortality in the first month after heart failure diagnosis is reported as n (%), with the denominator being the number of people diagnosed during that time period. Estimates have 95% CIs in parentheses. With the exception of age groups, all predictions are estimated at the mean population age (77·8 years). N/A=not applicable.

* Relative percentage difference in mortality (per 100 person-years) between the first and second diagnosis calendar time periods.

† p value for the difference in trend lines between groups.

‡ Average annual percentage change in rates (per 100 person-years) for each increasing year of diagnosis.

§ Change in slope estimated using Joinpoint regression.

¶ Among those who survived the first month after diagnosis.

‖ Among those who survived the first year after diagnosis.

During 1998–2001, age-adjusted first-year and subsequent-year rates of all-cause and cause-specific hospitalisations were higher for men than for women (Table 1, Table 2). However, by 2012–15, first-year rates for all-cause hospitalisations were significantly higher in women than in men (p<0·0001), explained by increasing rates for women and stable rates in men (table 2, figure 1). Also, the first-year rates of non-cardiovascular disease hospitalisation increased twice as much among women (3·9% per annum) than among men (1·4% per annum; table 2). Similar patterns were found for subsequent-year rates (appendix pp 9–11). After full covariate adjustment, women had a lower IRR for all cause-specific hospitalisation than men did, with the largest decrease seen for cardiovascular disease hospitalisations (table 4). However, when stratified by year of diagnosis, these risk differences lessened and became non-significant in later years (data not shown).

**Table 4:** Association between population group and hospitalisation

		**Total admissions**	**Follow-up, person-years**		**Cause of hospitalisation**					
			Total	Median (IQR)	All-cause		Cardiovascular disease		Non-cardiovascular disease	
					Unadjusted IRR	Adjusted IRR*	Unadjusted IRR	Adjusted IRR*	Unadjusted IRR	Adjusted IRR*
All		228 113	330 137	2·4 (0·5–5·7)	··	··	··	··	··	··
Age, years										
	<70	57 164	106 267	4·8 (1·6–9·2)	1·0 (ref)	1·0 (ref)	1·0 (ref)	1·0 (ref)	1·0	1·0
	70–79	77 526	115 053	3·3 (0·9–6·9)	1·31 (1·27–1·34)	1·21 (1·18–1·24)	1·29 (1·24–1·34)	1·18 (1·13–1·23)	1·31 (1·27–1·35)	1·23 (1·20–1·27)
	80–89	76 565	91 903	1·8 (0·3–4·2)	1·64 (1·60–1·68)	1·47 (1·43–1·51)	1·65 (1·59–1·71)	1·45 (1·39–1·52)	1·64 (1·59–1·68)	1·49 (1·45–1·54)
	≥90	16 858	16 914	0·7 (0·1–2·3)	1·94 (1·87–2·01)	1·72 (1·65–1·79)	1·89 (1·79–1·99)	1·67 (1·57–1·77)	1·95 (1·88–2·03)	1·74 (1·67–1·82)
Sex										
	Male	116 829	174 160	2·5 (0·5–5·9)	1·0 (ref)	1·0 (ref)	1·0 (ref)	1·0 (ref)	1·0 (ref)	1·0 (ref)
	Female	111 284	155 977	2·2 (0·4–5·4)	1·05 (1·03–1·07)	0·91 (0·90–0·92)	0·97 (0·94–1·00)	0·85 (0·83–0·88)	1·09 (1·07–1·11)	0·90 (0·88–0·92)
Socioeconomic status										
	Quintile 1 (most affluent)	37 567	62 395	2·4 (0·5–5·8)	1·0 (ref)	1·0 (ref)	1·0 (ref)	1·0 (ref)	1·0 (ref)	1·0 (ref)
	Quintile 2	50 412	76 813	2·4 (0·5–5·7)	1·08 (1·05–1·11)	1·05 (1·02–1·08)	1·10 (1·05–1·15)	1·06 (1·02–1·11)	1·08 (1·04–1·11)	1·05 (1·02–1·08)
	Quintile 3	47 541	70 313	2·3 (0·4–5·7)	1·13 (1·10–1·17)	1·10 (1·07–1·13)	1·14 (1·09–1·19)	1·10 (1·05–1·15)	1·13 (1·09–1·17)	1·09 (1·06–1·12)
	Quintile 4	48 043	65 182	2·4 (0·4–5·6)	1·20 (1·16–1·23)	1·14 (1·11–1·18)	1·16 (1·11–1·22)	1·12 (1·08–1·17)	1·22 (1·18–1·26)	1·16 (1·12–1·19)
	Quintile 5 (most deprived)	44 228	54 986	2·3 (0·4–5·6)	1·34 (1·32–1·35)	1·20 (1·17–1·24)	1·23 (1·17–1·29)	1·18 (1·13–1·19)	1·33 (1·28–1·37)	1·22 (1·21–1·25)
Place of diagnosis										
	Community	135 050	234 304	3·3 (0·9–6·9)	1·0 (ref)	1·0 (ref)	1·0 (ref)	1·0 (ref)	1·0 (ref)	1·0 (ref)
	Hospital	93 063	95 833	1·3 (0·2–3·8)	1·76 (1·73–1·80)	1·52 (1·49–1·55)	2·04 (1·98–2·10)	1·77 (1·72–1·82)	1·64 (1·63–1·66)	1·40 (1·37–1·43)

Data are n, person-years, median (IQR), or IRR (95% CI). IRR=incidence rate ratio.

* Adjusted for all covariates.

In 1998–2001, first-year rates of mortality were higher for men than for women but the gap between the sexes decreased by 2012–15 (table 3). This decrease in the gap was due to a 2·6% per annum decrease in mortality for men until 2009, which stabilised thereafter, compared with stable mortality for women throughout the study period (table 3, figure 2). Subsequent-year rates of mortality decreased at twice the rate in men (–2·2% per annum) as in women (–0·8% per annum; table 3). Overall, age-standardised mortality risk at 1, 3, and 5 years after diagnosis was approximately 3–4% higher in men than in women, which persisted throughout the study period (appendix p 12). Before adjustment, mortality risk was higher in women than in men; however, after full adjustment, women were at lower risk than men of all-cause mortality, and cardiovascular disease and non-cardiovascular disease deaths (table 5; appendix p 4). Again, after stratifying by year of diagnosis, the difference between the sexes was attenuated, becoming non-significant by 2016 (data not shown).

**Table 5:** Associations between population groups and mortality

		**Total deaths**	**Mean survival time, years**	**Median age at death, years**	**Cause of death**					
					All-cause		Cardiovascular disease		Non-cardiovascular disease	
					Unadjusted HR	Adjusted HR*	Unadjusted HR	Adjusted HR*	Unadjusted HR	Adjusted HR*
All		65 063 (74%)	3·2 (3·1–3·2)	84 (78–90)	··	··	··	··	··	··
Age, years										
	<70	8859 (49%)	9·0 (8·8–9·3)	67 (62–71)	1·0 (ref)	1·0 (ref)	1·0 (ref)	1·0 (ref)	1·0 (ref)	1·0 (ref)
	70–79	18 517 (72%)	4·5 (4·5–4·6)	79 (76–82)	1·88 (1·84–1·93)	1·68 (1·64–1·73)	1·77 (1·70–1·83)	1·60 (1·54–1·66)	2·00 (1·93–2·08)	1·73 (1·66–1·81)
	80–89	27 568 (83%)	2·2 (2·1–2·2)	87 (84–89)	3·27 (3·19–3·36)	2·66 (2·58–2·74)	3·15 (3·04–3·26)	2·59 (2·49–2·70)	3·33 (3·20–3·46)	2·61 (2·49–2·73)
	≥90	10 119 (90%)	0·8 (0·8–0·9)	94 (92–96)	5·59 (5·42–5·76)	4·08 (3·93–4·22)	5·30 (5·08–5·52)	3·95 (3·76–4·15)	5·84 (5·57–6·12)	4·05 (3·82–4·29)
Sex										
	Male	31 819 (71%)	3·5 (3·4–3·5)	82 (75–88)	1·0 (ref)	1·0 (ref)	1·0 (ref)	1·0 (ref)	1·0 (ref)	1·0 (ref)
	Female	33 244 (76%)	2·9 (2·8–2·9)	86 (80–91)	1·15 (1·13–1·17)	0·85 (0·83–0·86)	1·11 (1·09–1·13)	0·85 (0·83–0·88)	1·21 (1·18–1·24)	0·84 (0·82–0·87)
Socioeconomic status										
	Quintile 1 (most affluent)	11 645 (71%)	3·4 (3·3–3·5)	86 (80–90)	1·0 (ref)	1·0 (ref)	1·0 (ref)	1·0 (ref)	1·0 (ref)	1·0 (ref)
	Quintile 2	15 093 (74%)	3·2 (3·3–3·5)	85 (79–90)	1·05 (1·03–1·08)	1·05 (1·03–1·08)	1·07 (1·04–1·11)	1·09 (1·05–1·12)	1·02 (0·98–1·06)	1·01 (0·97–1·05)
	Quintile 3	14 065 (74%)	3·1 (3·0–3·2)	85 (89–90)	1·07 (1·04–1·10)	1·07 (1·05–1·10)	1·11 (1·07–1·15)	1·12 (1·09–1·16)	1·03 (1·00–1·08)	1·02 (0·98–1·06)
	Quintile 4	12 971 (74%)	3·1 (3·0–3·2)	84 (77–89)	1·06 (1·04–1·09)	1·11 (1·09–1·14)	1·06 (1·02–1·10)	1·12 (1·09–1·17)	1·06 (1·02–1·11)	1·10 (1·06–1·14)
	Quintile 5 (most deprived)	11 149 (75%)	3·1 (2·9–3·2)	82 (75–88)	1·08 (1·05–1·11)	1·17 (1·14–1·21)	1·06 (1·02–1·10)	1·18 (1·14–1·23)	1·12 (1·07–1·16)	1·17 (1·13–1·22)
Place of diagnosis										
	Community	37 734 (73%)	4·2 (4·2–4·3)	84 (78–90)	1·0 (ref)	1·0 (ref)	1·0 (ref)	1·0 (ref)	1·0 (ref)	1·0 (ref)
	Hospital	27 329 (75%)	1·9 (1·8–1·9)	85 (78–90)	1·55 (1·53–1·58)	1·46 (1·43–1·48)	1·72 (1·69–1·76)	1·55 (1·51–1·59)	1·28 (1·25–1·31)	1·37 (1·34–1·40)

Data are n (%), mean (95% CI), median (IQR), and HR (95% CI). HR=hazard ratio.

* Adjusted for all covariates.

In 1998–2001, first-year rates of any-cause hospitalisation were significantly higher in the most deprived socioeconomic group (quintile 5) than in the most affluent group (quintile 1; table 2). This difference persisted over time due to similar increasing rates of hospitalisation in both groups (p=0·103 for interaction of trend lines; table 2, figure 1) and subsequent-year rates showed a similar pattern (appendix pp 9–11). In the most recent time period (2012–15), the biggest difference between the most affluent and most deprived groups was for first-year rate of hospitalisation for non-cardiovascular disease (table 2). In the fully adjusted model, the most deprived group had a significantly higher risk than the most affluent group of all-cause, cardiovascular disease, and non-cardiovascular disease hospitalisation (table 4). Risk increased with quintiles of socioeconomic status from the highest (quintile 1) to the lowest quintile (quintile 5; table 4).

In 2012–15, first-year rates of mortality were higher in the most deprived group than in the most affluent group (table 3, figure 2). Age-standardised mortality risk increased for each quintile of socioeconomic status, from the most affluent to the most deprived group: in 2012–15, for quintile 1 versus quintile 5, mortality risk was 27% versus 32% at 1 year after diagnosis, 43% versus 50% at 3 years after diagnosis, and 56% versus 63% at 5 years after diagnosis (appendix p 12). After full adjustment, the most deprived group still had a higher risk than the most affluent group of all-cause, cardiovascular disease, and non-cardiovascular disease death (table 5), and age-standardised survival decreased for each quintile of socioeconomic status (appendix p 4).

First-year rates of any-cause hospitalisation were significantly higher in the hospital-diagnosed group than in the community-diagnosed group, with similar growth over time (table 2). Annual rates of admission between the two groups began to diverge after 2004, decreasing at the same rate for the community group as they increased for the hospital group (–1·3% and 0·9% per annum; table 2, figure 1). The hospital group had a consistent 2·4% per annum increase in first-year rate of non-cardiovascular disease hospitalisation, whereas first-year rates did not increase for the community group after 2004 (table 2). When adjusted for all covariates, the hospital-diagnosed group had significantly higher risk than the community-diagnosed group of hospitalisation due to all-causes, cardiovascular disease and non-cardiovascular disease (table 4).

First-year rates of mortality in the hospital-diagnosed group were nearly double the rates in the community-diagnosed group in 1998–2001, and this gap widened over time to 2012–15 (p<0·0001). Age-adjusted first-year rates of mortality decreased in the community-diagnosed group (–2·7% per annum) at twice the rate of the hospital-diagnosed group (–1·1% per annum; table 3, figure 2). A similar pattern was identified for subsequent-year rates (table 3). Over the study period, first-year mortality risk in the hospital-diagnosed group was 36% whereas it was 27% in the community-diagnosed group. During 2012–15, age-standardised mortality risk remained significantly higher in the hospital-diagnosed group than in the community-diagnosed group: 1 year (34% *vs* 24%), 3 years (53% *vs* 40%), and 5 years after diagnosis (66% *vs* 52%; appendix p 12). Overall, median survival time in the community-diagnosed group was twice that of the hospital-diagnosed group (table 5; appendix p 4). In the fully adjusted models, the hospital-diagnosed group had significantly higher risk than the community-diagnosed group of all-cause, cardiovascular disease, and non-cardiovascular disease deaths (table 5).

For all groups, risk of cardiovascular death was higher than non-cardiovascular disease deaths for the first few years after diagnosis of heart failure, but the two causes converged in later years with non-cardiovascular disease causes of death being the most common cause of death at around 3–5 years (appendix p 5). In our sensitivity analysis, effects remained relatively stable after full-case analysis (appendix p 13).

## Discussion

Our study provides a comprehensive and contemporary assessment of outcomes in patients with incident heart failure who were diagnosed with heart failure in hospital and community settings. In a large, nationally representative sample over 20 years, we identified specific diverging trends and inequalities in admission to hospital and mortality in the UK. Non-elective hospitalisation burden has increased, particularly for non-cardiovascular disease causes, which account for over two-thirds of all admissions. Heart failure remains associated with a high risk of death, with only minimal improvement in risk over the past 20 years. Inequalities and poor outcomes have persisted, particularly for the oldest age groups, men, the most deprived, and for patients diagnosed in hospital, and new worrying trends for women have emerged.

Mortality risk after a diagnosis of heart failure has decreased slightly over the past two decades; whereas 1-year and 5-year mortality risk has decreased by 3–4%. This small decrease is probably in part because of advances in diagnostic technology to identify milder heart failure. Despite the decrease in risk, our 1-year and 5-year figures are similar or worse than those in 2000 in the Framingham^23^ (29% at 1 year) and Rochester^24^ (48% at 5 years) cohorts. Hospital cohorts in the USA,^11^ Scotland,^25^ and Demark^26^ reported slightly lower 1-year risks (29% in the USA, 33% in Scotland, and 30% in Denmark) than the 34% risk in our hospital-diagnosed group. Similarly, community cohorts from the UK^14^ have reported 1-year mortality risk of 24% compared with the 27% overall 1-year mortality risk in our community-diagnosed group. Like most epidemiological evidence on heart failure from hospital-only or primary care-only cohorts, these cohort studies were not able to ascertain the date of heart failure diagnosis. By linking community and hospital records, we were able to identify the date of incident heart failure, which probably accounts for the higher risk of mortality in our study and indicates that the true risk burden for patients with heart failure might be higher than previously reported. The persisting high risk associated with heart failure possibly reflects the ageing multimorbid population, alongside a survival plateau in the era of effective, evidence-based treatment for heart failure (selective β blockers and angiotensin-converting enzyme inhibitors). The increase in the number of admissions to hospital due to non-cardiovascular disease events and that 50% of deaths were due to non-cardiovascular disease events would support this hypothesis.

We observed a new and important pattern of increasing hospitalisation due to heart failure. Previous evidence on cause-specific patterns grouped admissions due to heart failure with cardiovascular admissions, reporting stable or decreasing rates over time.^5^ By partitioning out heart failure from other cardiovascular admissions, we found a new pattern that has not previously been reported. This finding is particularly important given the increase in the number of outpatient units that should reduce the number of patients with acute heart failure who need to be admitted to hospital, meaning that our findings might underestimate the worsening problem of acute heart failure. Increased hospitalisation due to heart failure was particularly pronounced in women and possibly indicates later presentation of more severe heart failure in older multimorbid patients than would be seen in men. Other potential triggers might be a lack of evidence-based therapies for heart failure with preserved ejection fraction, which is increasing in prevalence,^27^ or improved survival of patients with severe heart failure with contemporary therapies for heart failure.

Patients who had hospital-diagnosed heart failure had significantly worse outcome rates over time compared with the community-diagnosed group; a finding shared by previous reports.^28^, ^29^ Our findings reveal an exponential increase in the number of patients diagnosed with heart failure in hospital, accounting for 74% of the population admitted for heart failure by 2017. Furthermore, our study showed that the steeply increasing burden of non-cardiovascular disease hospitalisations were predominantly in the hospital-diagnosed group. Mortality was also significantly higher for the hospital-diagnosed group than for the community-diagnosed group, with slower rates of decrease than in the community-diagnosed group. Delayed diagnosis, higher comorbidity burden, and lower pres-cribing of first-line preventive medications, leading to more severe heart failure at the point of diagnosis, might in part explain the worse prognosis in this group than in the community-diagnosed group. The 2018 UK National Heart Failure Audit^30^ suggests that an increase in dedicated heart failure specialists results in better outcomes for patients who have been admitted to hospital and policy recommends that all patients with heart failure have access to a multidisciplinary team.^31^ Notably, evidence-based treatments for heart failure are relevant only to those with decreased ejection fraction, and some of the apparent under-use of these treatments might be explained by a lack of evidence for the use of these treatments in patients with heart failure with preserved ejection fraction. A 2018 report showed that around half of patients who are diagnosed in hospital present earlier to their primary care physician with heart failure symptoms,^32^ indicating that an urgent need exists for specialist support for earlier diagnosis.

Previous heart failure studies have shown worse outcomes for men than women.^6^, ^33^ Our findings show that faster increases in rates of admission to hospital due to heart failure and non-cardiovascular disease and slower decreases in mortality in women than men has resulted in similar outcomes between the sexes over the past decade; a finding also reported in Denmark^26^ and Ontario.^28^ These patterns likely reflect the increasing prevalence of non-cardiovascular disease comorbidities in women. However, the attenuation of sex differences over time, even after accounting for comorbidities, and a greater increase in the rate of hospitalisations due to heart failure over time in women than in men suggest worsening severity of heart failure or a lack of effective therapies for heart failure in women compared with men. These unfavourable patterns in women might also be associated with the increasing prevalence of heart failure with preserved ejection fraction in women (for which, to date, no proven therapy exists to improve outcomes).^12^, ^34^

The most deprived group had consistently worse outcomes than the most affluent group, with 20% higher risk of all-cause hospitalisation even after adjustment; an inequality that persisted over the past 20 years. Previously, socioeconomic status has not been associated with cause-specific hospitalisation,^7^ but our study highlights a new finding of increased risk across all cause-specific outcomes in the most deprived patients with heart failure. Although increased burden of comorbidities and worse lifestyle risk factors might partly explain the increased risk in this group, the persisting difference after full adjustment for these factors suggests other social and health-care factors might be having an effect.^35^ Potential delays before seeking help,^36^ with increased severity of comorbidities and heart failure at the point of diagnosis, might be one explanation and points to the preventable nature of heart failure. Lower prevalence of cancer in the most deprived group than in the most affluent group opposes the patterns seen for other comorbidities in our population but is perhaps unsurprising considering persistent socioeconomic inequalities in cancer survival.^37^

To our knowledge, this is the largest study of incident heart failure to report trends in cause-specific hospitalisation and mortality in general population groups. By linking large, nationally representative databases, we were able to ascertain the incident date of heart failure and follow up patients for up to 20 years for cause-specific hospitalisation and death. However, the use of routinely collected data means diagnosis of heart failure can be subject to misclassification and measurement error, which might change over time. Diagnosis of heart failure in the community is clinically defined and we cannot rule out that the lower risk of all hospitalisation and mortality outcomes in the community-diagnosed group might in part be due to misclassification. However, in the UK, diagnosis of heart failure has been enhanced by the introduction of echocardiography as part of performance incentives^38^ and national heart failure audits and heart failure diagnoses have been shown to have high precision in CPRD.^17^ The outcomes measured in this study are also dependent on severity or type of heart failure but routine data did not provide echocardiographic data or specific heart failure phenotypes. To account for severity, a range of comorbidity and medications were included in our analyses, but further investigation on specific phenotypes of heart failure with outcomes is required. Accuracy of clinical recording and diagnoses in the CPRD have been found to be valid for a range of morbidities^16^ and we also used primary ICD codes for cause-specific outcomes and clinically validated code sets, which have high precision.^17^ Changes in diagnostic procedures over time, such as advanced echocardiography to detect milder forms of heart failure, might have influenced our findings and be partly responsible for the small decrease in mortality risk over time. This bias would only serve to lessen the overall mortality risk in the later time period and stem increasing rates of hospitalisation, such that the overall burden of heart failure could be even higher than reported here.

Population inequalities in outcomes of heart failure are a major and persistent public health challenge. Diverging trends among groups suggests that disparities according to age, sex, socioeconomic status, and place of diagnosis are likely to increase. Tailored management strategies that include access to teams specialising in heart failure together with social interventions are urgently required to address the growing complexity of patients with heart failure and decrease the inequitable outcomes of care.

## Data sharing

Data access is through permission from Clinical Practice Research Datalink only; please send any enquiries to isac@cprd.com.
