# Supplementary material for: 20-year trends in cause-specific heart failure outcomes by sex, socioeconomic status, and place of diagnosis: a population-based study
Source: Lancet Public Health. 2019 Jul 31;4(8):e406–20. doi: 10.1016/S2468-2667(19)30108-2 (PMC6686076; doi:10.1016/S2468-2667(19)30108-2)
Supplement: Supplementary appendix [file mmc1.pdf]

THE LANCET
Public Health

Supplementary appendix

This appendix formed part of the original submission and has been peer reviewed. We post it as supplied by the authors.

Supplement to: Lawson CA, Zaccardi F, Squire I, et al. 20-year trends in cause-specific heart failure outcomes by sex, socioeconomic status, and place of diagnosis: a population-based study. *Lancet Public Health* 2019; **4:** e406–20.

SUPPLEMENTARY MATERIAL

Health inequalities in heart failure; national trends in outcomes by place of diagnosis, gender and socioeconomic status: 1998-2017

Lawson CA, Zaccardi F, Squire I, Ling S, Davies M, Lam C, Mamas M, Khunti K, Kadam UT

Table of Contents

Supplementary Figures 2

S1 Flow chart of the patient selection process 2

S2 Proportion of HF patients diagnosed in the community or hospital by calendar year 3

S3 Predicted overall survival by population group 4

S4 Cumulative incidence of death by cause and population group 5

Supplementary Tables 6

S1 Heart failure selection CPRD code set 6

S2 Heart failure selection ICD code set 7

S3 Missing data 8

S4 Predicted rates of admissions during the second and subsequent years following HF diagnosis by population groups and calendar year 9

S5 Age standardised mortality risk by diagnosis time-period and population group at 1, 3 and 5 years following HF diagnosis 12

S6 Complete case analysis: Associations between population groups 13

Supplementary Figures

S1 Flow chart of the patient selection process

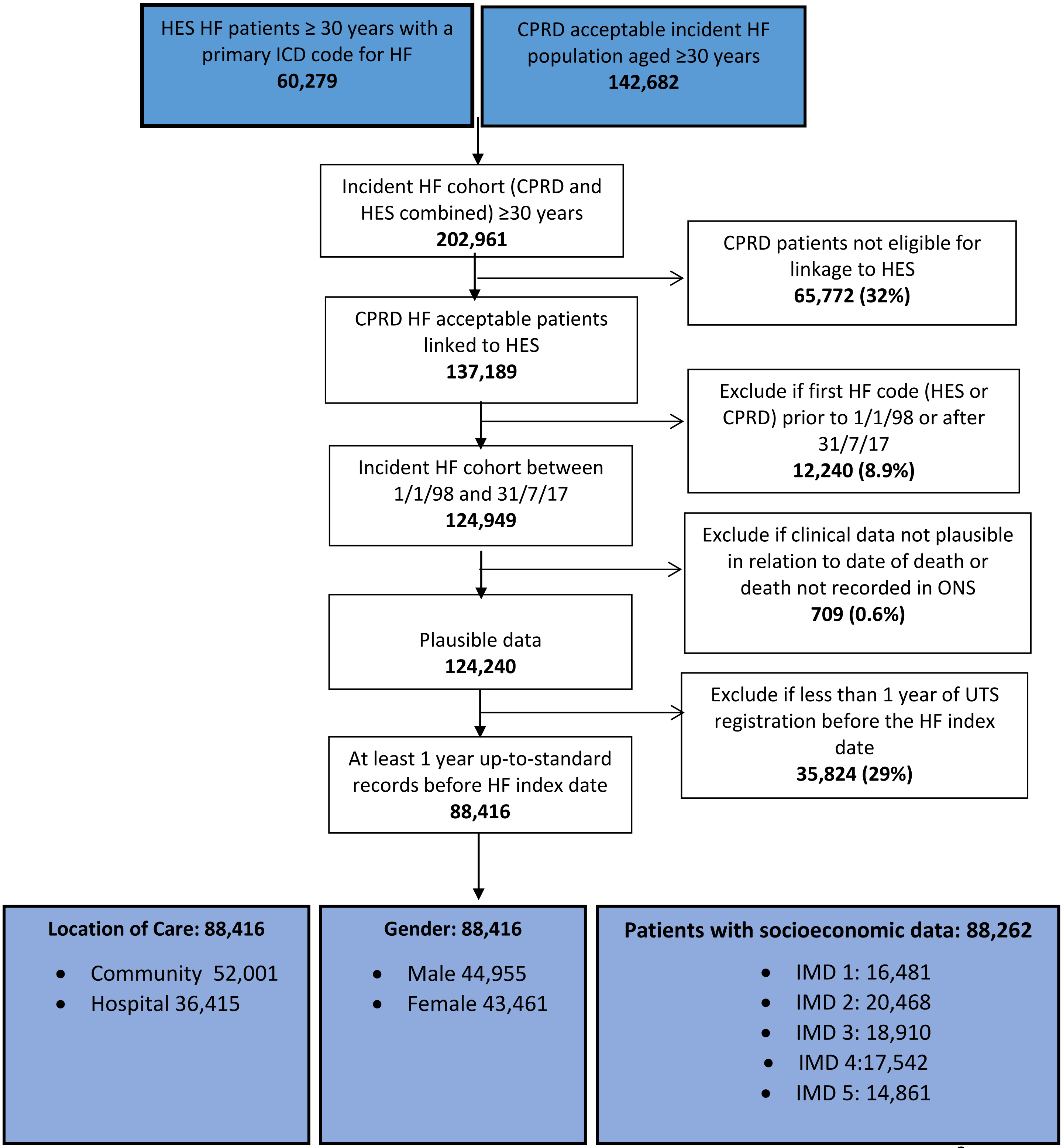

S2 Proportion of HF patients diagnosed in the community or hospital by calendar year

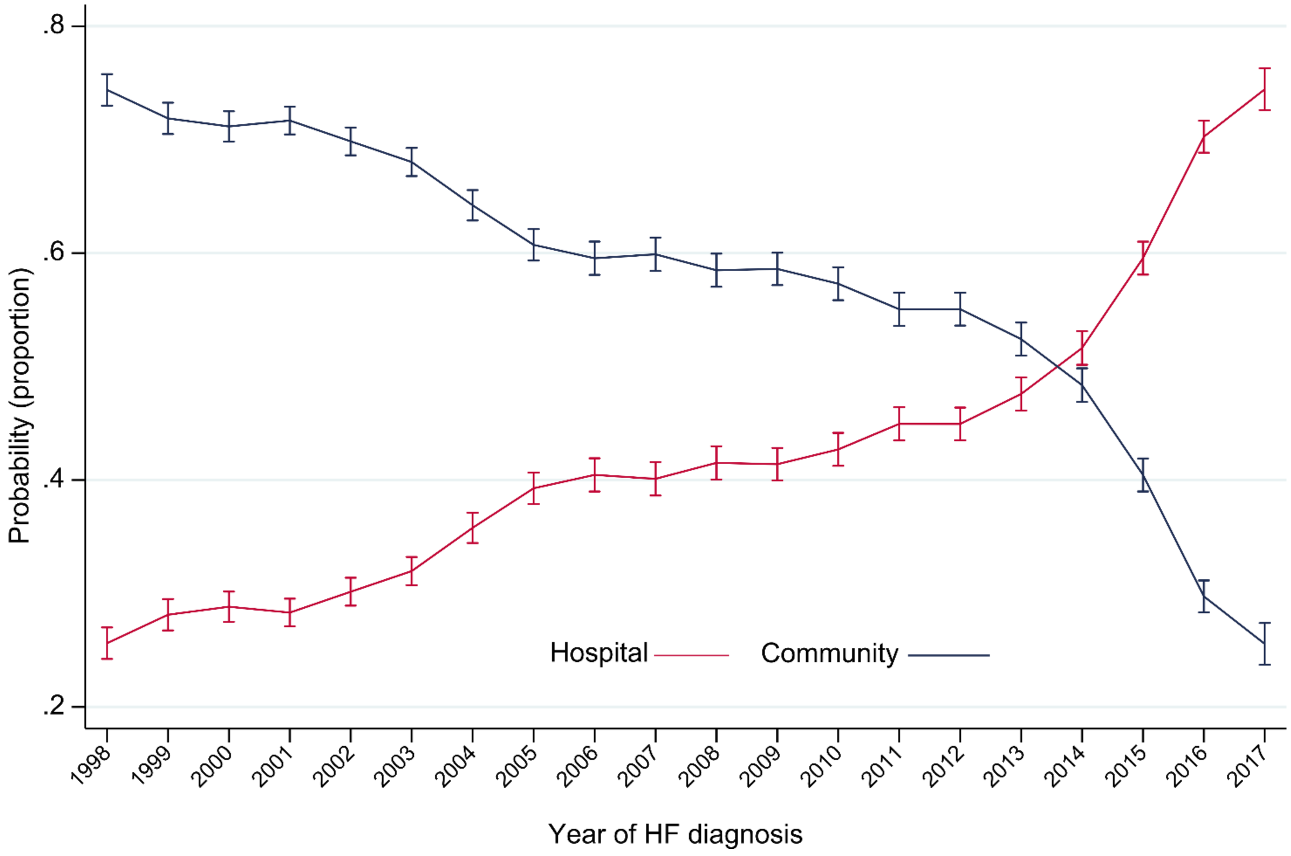

S3 Predicted overall survival by population group

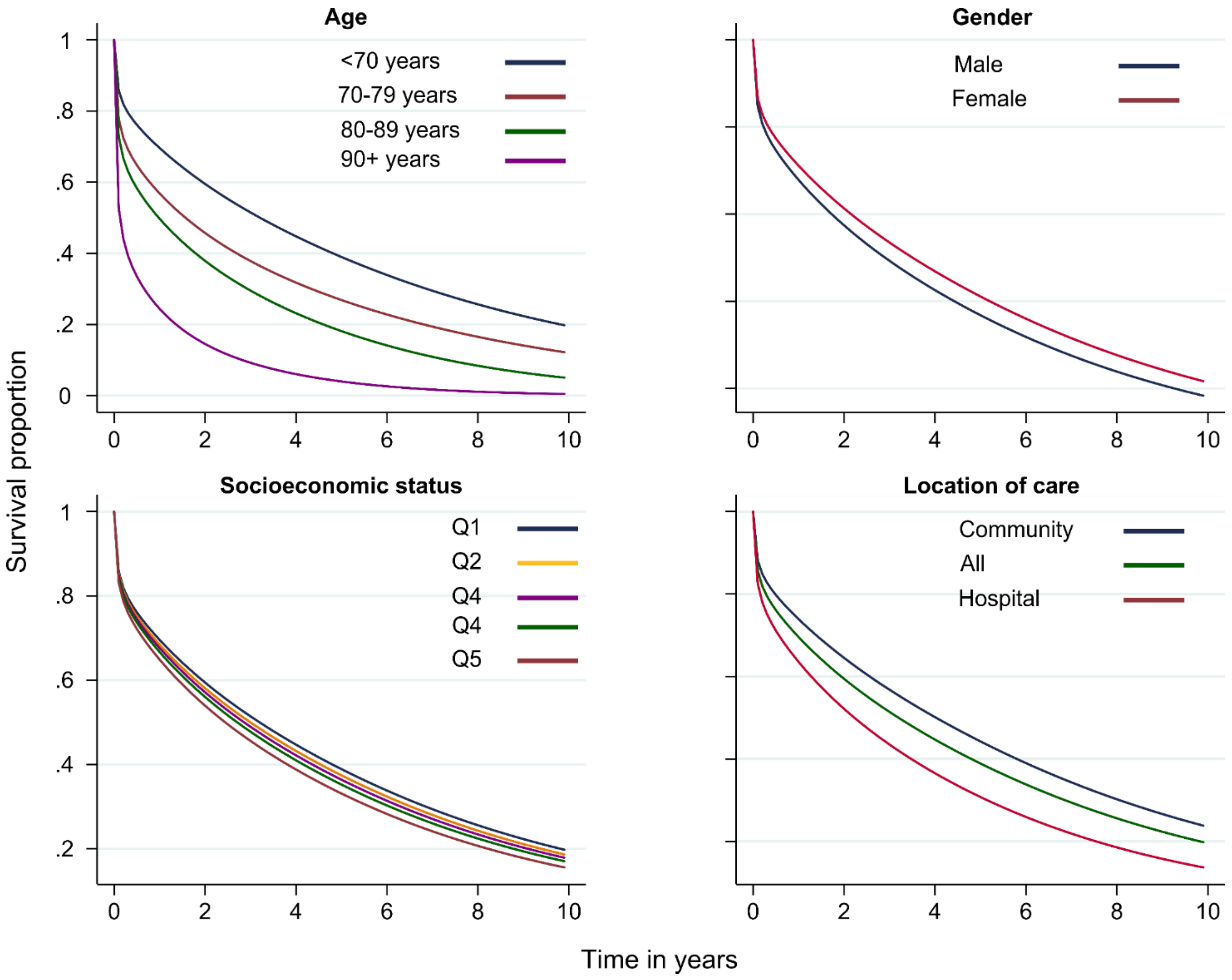

Age-standardised survival curves plotted against time in years following HF diagnosi

S4 Cumulative incidence of death by cause and population group

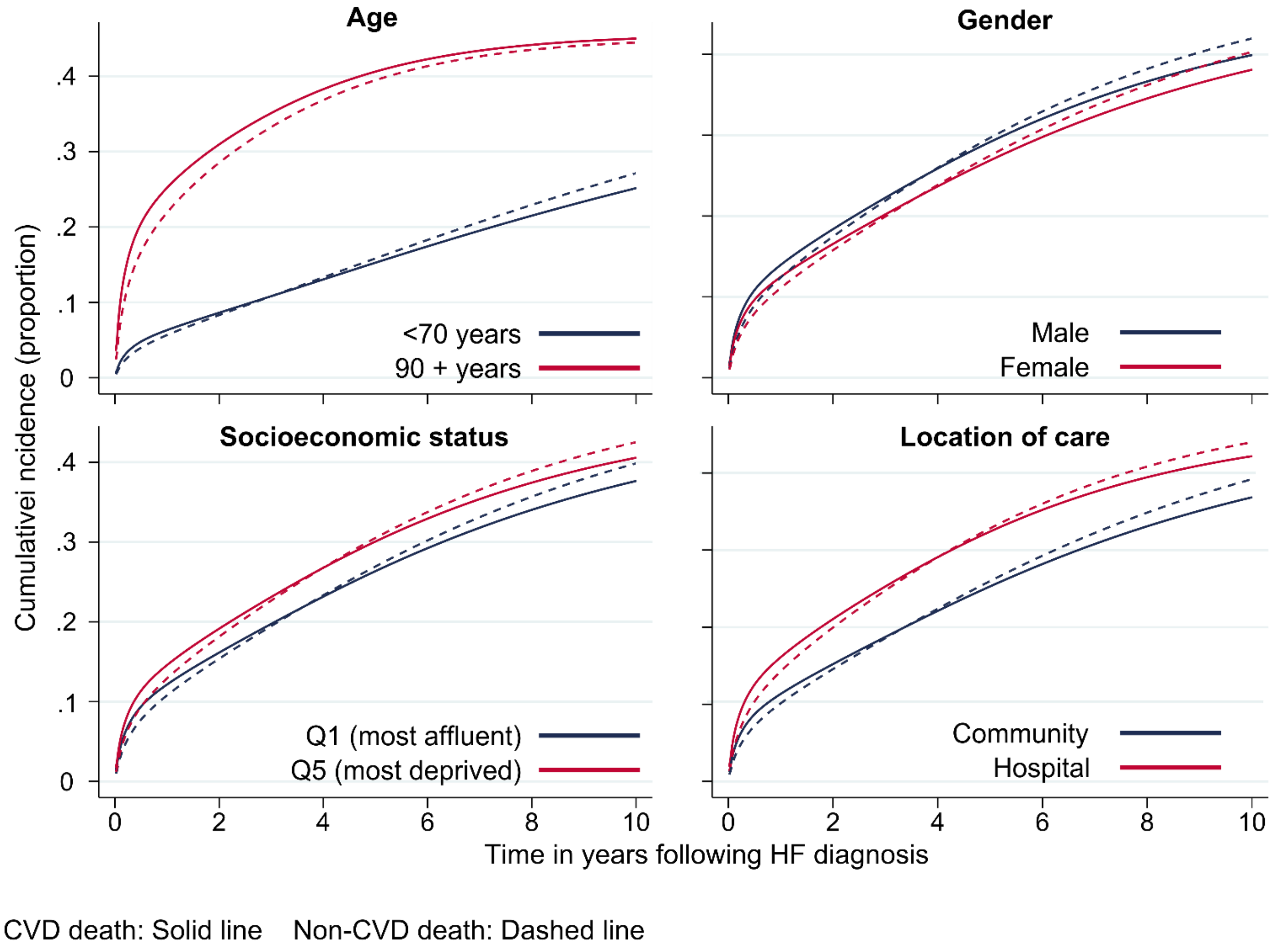

Cumulative incidence curves predicted at mean population age (78 years).

Supplementary Tables

S1 Heart failure selection CPRD code set

Medcode	Read code	Read term
2062	G58..00	Heart failure
1223	G58..11	Cardiac failure
398	G580.00	Congestive heart failure
2906	G580.11	Congestive cardiac failure
10079	G580.12	Right heart failure
10154	G580.13	Right ventricular failure
9524	G580.14	Biventricular failure
23707	G580000	Acute congestive heart failure
32671	G580100	Chronic congestive heart failure
27884	G580200	Decompensated cardiac failure
11424	G580300	Compensated cardiac failure
94870	G580400	Congestive heart failure due to valvular disease
884	G581.00	Left ventricular failure
23481	G581.11	Asthma - cardiac
43618	G581.12	Pulmonary oedema - acute
5942	G581.13	Impaired left ventricular function
5255	G581000	Acute left ventricular failure
27964	G582.00	Acute heart failure
101138	G583.00	Heart failure with normal ejection fraction
101137	G583.11	HFNEF - heart failure with normal ejection fraction
106897	G583.12	Heart failure with preserved ejection fraction
104275	G584.00	Right ventricular failure
4024	G58z.00	Heart failure NOS
12590	G58z.11	Weak heart
17278	G58z.12	Cardiac failure NOS
9913	1O1..00	Heart failure confirmed
22262	G1yz100	Rheumatic left ventricular failure
21837	G232.00	Hypertensive heart&renal dis wth (congestive) heart failure

S2 Heart failure selection ICD code set

ICD-10 Code	ICD-10 term
I50	Heart failure
150.0	Congestive heart failure
I50.1	Left ventricular failure
I50.9	Heart failure, unspecified
I11.0	Hypertensive heart disease with (congestive) heart failure
I13.2	Hypertensive heart and renal disease with both (congestive) heart failure and renal failure
I13.0	Hypertensive heart and renal disease with (congestive) heart failure

S3 Missing data

Characteristics	Missing (%)
Age	-
Gender	-
Socioeconomic status	0.2
Prescribed drugs	17.5
Comorbidities	-
Smoking	5.9
Alcohol	14.1
BMI (kg/m2)	14.7
Systolic BP (mm/Hg)	2.6
Cholesterol (mmol/L)	32.9
Haemoglobin (g/dL)	24.7
eGFR (ml/min/m2)	23.3

BMI, body mass index; BP, blood pressure; eGFR, estimated glomerular filtration rate

S4 Predicted rates of admissions during the second and subsequent years following HF diagnosis by population groups and calendar year

	Predicted rate per 100 person-years (95% CI)		Relative diff. (%)[a]	P Interaction[b]	Average annual percent change per year (95% CI)[c]	Slope change: annual percent change before and after trend change (95% CI)[d]		
	1998-2001	2012-2015				Before	Break	After
**All-cause admissions**								
All	81.6 (79.7, 83.5)	82.9 (80.8, 85.0)	2		0.2 (-0.6, 0.9)	0.2 (-0.6, 0.9)	N/A	0.2 (-0.6, 0.9)
<70 years	69.2 (65.9, 72.5)	53.3 (50.7, 56.0)	-23		-1.5 (-2.8, -0.1)			
70 to 79 years	77.8 (74.9, 80.8)	76.4 (72.9, 79.9)	-2	<0·0001	-0.3 (-1.2, 0.5)	2.1 (0.9, 3.3)	2007	-3.0 (-4.4, -1.5)
80 to 89 years	85.7 (82.1, 89.3)	99.4 (95.3, 103.5)	16	<0·0001	1.1 (0.1, 2.1)	3.8 (2.3, 5.3)	2007	-1.9 (-3.3, -0.4)
90 years and over	82.1 (74.3, 89.9)	121.7 (111.8, 131.6)	48	<0·0001	3.2 (1.6, 4.7)	6.3 (3.3, 9.4)	2006	0.4 (-1.3, 2.3)
Men	88.3 (85.3, 91.3)	81.0 (78.2, 83.7)	-8		-0.4 (-1.1, 0.3	-0.4 (-1.1, 0.3	N/A	-0.4 (-1.1, 0.3
Women	75.8 (73.4, 78.3)	85.3 (82.2, 88.4)	13	<0·0001	0.7 (-0.8, 2.1)	0.7 (-0.8, 2.1)	N/A	0.7 (-0.8, 2.1)
Most affluent (Q1)	69.0 (65.0, 73.0)	69.6 (65.6, 73.5)	1		0.2 (-1.3, 1.7)	3.3 (-0.5, 7.4)	2004	-1.5 (-2.9, 0.0)
Most deprived (Q2)	99.0 (93.6, 104.3)	101.9 (95.6, 108.1)	3	0·113	0.4 (-1.1, 1.8)	0.4 (-1.1, 1.8)	N/A	0.4 (-1.1, 1.8)
Community diagnosis	72.7 (70.7, 74.6)	65.9 (63.7, 68.1)	-9		-1.1 (-2.0, -0.2)	0.5 (-0.3, 1.4)	2009	-4.0 (-6.2, -1.8)
Hospital diagnosis	112.0 (106.7, 117.3)	106.5 (102.6, 110.4)	-5	0·262	-0.1 (-0.9, 0.8)	3.2 (1.7, 4.8)	2006	-2.9 (-4.1, -1.8)
**HF admissions**								
All	8.8 (8.3, 9.3)	9.7 (9.1, 10.3)	10		0.6 (-0.1, 1.4)	0.6 (-0.1, 1.4)	N/A	0.6 (-0.1, 1.4)
<70	7.7 (6.9, 8.6)	5.6 (4.9, 6.3)	-27		-2.7 (-3.9, -1.5)	-2.7 (-3.9, -1.5)	N/A	-2.7 (-3.9, -1.5)
70 to 79	8.0 (7.3, 8.7)	9.5 (8.4, 10.5)	19	<0·0001	0.8 (-0.2, 1.7)	0.8 (-0.2, 1.7)	N/A	0.8 (-0.2, 1.7)
80 to 89	9.1 (8.2, 10.0)	12.0 (10.8, 13.1)	32	<0·0001	1.9 (0.2, 3.5)	1.9 (0.2, 3.5)	N/A	1.9 (0.2, 3.5)
>90	7.2 (5.4, 9.0)	12.3 (9.8, 14.9)	71	<0·0001	1.7 (-2.6, 6.1)	6.0 (3.2, 8.9)	2012	-16.3 (-33.8, 5.7)
Men	10.4 (9.5, 11.2)	9.9 (9.1, 10.7)	-5		-0.5 (-1.3, 0.3)	-0.5 (-1.3, 0.3)	N/A	-0.5 (-1.3, 0.3)
Women	7.4 (6.9, 8.0)	9.5 (8.6, 10.3)	28	<0·0001	1.5 (0.3, 2.7)	1.5 (0.3, 2.7)	N/A	1.5 (0.3, 2.7)
Most affluent	6.9 (6.0, 7.9)	8.8 (7.7, 10.0)	28		1.2 (-0.1, 2.5)	1.2 (-0.1, 2.5)	N/A	1.2 (-0.1, 2.5)

Most deprived	10.2 (9.0, 11.5)	11.9 (10.2, 13.6)	17	0·582	0.7 (-0.4, 1.7)	0.7 (-0.4, 1.7)	N/A	0.7 (-0.4, 1.7)
Community diagnosis	6.4 (6.0, 6.8)	6.5 (6.0, 7.1)	2		0.0 (-1.8, 1.9)	3.4 (-0.3, 7.3)	2005	-2.3 (-4.4, -0.1)
Hospital diagnosis	17.4 (15.6, 19.2)	14.2 (13.0, 15.4)	-18	0·040	-1.7 (-3.0, -0.4)	-1.7 (-3.0, -0.4)	N/A	-1.7 (-3.0, -0.4)
**Other CVD admissions**								
All	14.1 (13.6, 14.7)	11.7 (11.1, 12.2)	-17		-1.3 (-2.2, -0.4)	0.9 (-0.3, 2.1)	2007	-3.8 (-5.4, -2.2)
<70	12.8 (11.8, 13.7)	9.6 (8.8, 10.5)	-25		-1.9 (-2.7, -1.0)	-1.9 (-2.7, -1.0)	N/A	-1.9 (-2.7, -1.0)
70 to 79	13.8 (13.0, 14.7)	10.9 (10.0, 11.9)	-21	0.150	-1.2 (-2.7, 0.2)	-1.2 (-2.7, 0.2)	N/A	-1.2 (-2.7, 0.2)
80 to 89	14.2 (13.2, 15.2)	12.4 (11.4, 13.4)	-13	0.011	-1.1 (-2.7, 0.6)	2.9 (0.6, 5.2)	2007	-5.3 (-8.1, -2.5)
>90	13.6 (11.3, 15.9)	14.9 (12.5, 17.2)	10	<0.0001	1.4 (-0.5, 3.2)	1.4 (-0.5, 3.2)	N/A	1.4 (-0.5, 3.2)
Men	15.8 (14.9, 16.7)	12.0 (11.2, 12.7)	-24		-1.6 (-2.5, -0.8)		N/A	
Women	12.8 (12.1, 13.4)	11.3 (10.6, 12.1)	-12	0.003	-0.7 (-1.4, 0.0)	2.3 (1.1, 3.6)	2006	-3.3 (-4.4, -2.3)
Most affluent	12.3 (11.2, 13.4)	9.3 (8.3, 10.3)	-24		-1.8 (-3.3, -0.4)	-1.8 (-3.3, -0.4)	N/A	-1.8 (-3.3, -0.4)
Most deprived	15.7 (14.4, 17.1)	13.3 (11.7, 14.8)	-15	0·151	-0.8 (-2.1, 0.5)	-0.8 (-2.1, 0.5)	N/A	-0.8 (-2.1, 0.5)
Community diagnosis	12.5 (12.0, 13.1)	10.4 (9.8, 11.1)	-17		-1.6 (-2.7, -0.4)	-0.2 (-1.1, 0.7)	2010	-4.7 (-8.4, -0.8)
Hospital diagnosis	20.0 (18.5, 21.5)	13.4 (12.5, 14.3)	-33	<0.0001	-2.8 (-4.2, -1.3)	0.0 (-1.7, 1.8)	2007	-5.8 (-8.4, -3.1)
**Non CVD admissions**								
All	58.5 (57.0, 60.0)	64.0 (62.3, 65.8)	9		0.8 (0.1, 1.5)	3.6 ((2.4, 4.8)	2006	-1.6 (-2.5, -0.7)
<70	48.4 (45.9, 50.9)	39.7 (37.5, 41.8)	-18		-0.9 (-2.7, 0.9)	-0.9 (-2.7, 0.9)	N/A	-0.9 (-2.7, 0.9)
70 to 79	55.6 (53.4, 57.9)	58.3 (55.4, 61.2)	5	<0·0001	0.1 (-0.9, 1.2)	2.8 (1.3, 4.4)	2007	-2.8 (-4.6, -1.0)
80 to 89	62.3 (59.5, 65.1)	78.5 (75.0, 82.0)	26	<0·0001	1.7 (0.7, 2.8)	4.0 (2.2, 5.7)	2007	-0.7, -2.3, 0.9)
>90	60.7 (54.4, 67.0)	96.1 (87.7, 104.5)	58	<0·0001	3.0 (2.0, 4.0)	3.0 (2.0, 4.0)	N/A	3.0 (2.0, 4.0)
Men	62.1 (59.9, 64.4)	61.7 (59.5, 64.0)	-1		0.2 (-0.6, 1.0)	0.2 (-0.6, 1.0)	N/A	0.2 (-0.6, 1.0)
Women	55.3 (53.4, 57.2)	66.8 (64.1, 69.4)	21	<0·0001	1.0 (-0.7, 2.8)	1.0 (0.7, 2.8)	N/A	1.0 (0.7, 2.8)
Most affluent (Q1)	49.2 (46.1, 52.2)	53.8 (50.5, 57.1)	9		0.4 (-0.5, 1.3)	0.4 (-0.5, 1.3)	N/A	0.4 (-0.5, 1.3)
Most deprived (Q5)	73.5 (69.3, 77.8)	80.3 (75.0, 85.6)	9	0·200	0.8 (-0.9, 2.5)	0.8 (-0.9, 2.5)	N/A	0.8 (-0.9, 2.5)
Community diagnosis	53.8 (52.3, 55.4)	50.8 (49.0, 52.6)	-6		-0.8 (-1.7, 0.1)	0.7 (-0.2, 1.6)	2009	-3.5 (-5.7, -1.2)

Hospital diagnosis	74.2 (70.4, 78.0)	82.6 (79.3, 85.8)	11	<0·0001	1.1 (0.2, 2.0)	5.8 (4.2, 7.5)	2006	-2.9 (-4.0, -1.8)

Rates were conducted in survivors of the first year. With the exception of age groups, all predictions are at the mean population age (78 years). Follow up was until death or study end. Relative diff., relative difference; py, person-years; CI, confidence interval.
a relative percentage difference in admission rates (per 100 person-years) between the first and second diagnosis time periods, calculated by 100*([time-period 2 – time period 1] / time-period 1].
b P value for the difference in trend lines between groups. Estimated by fitting an interaction term between calendar year and group in the negative binomial models also containing age.
c Average annual percentage change in rates (per 100 person-years) for each increasing year of diagnosis
d Change in slope estimated using Joinpoint regression.

S5 Age standardised mortality risk by diagnosis time-period and population group at 1, 3 and 5 years following HF diagnosis

	1-year		3-year		5-year	
Calendar year of diagnosis	1998-2001	2012-2015	1998-2001	2012-2015	1998-2001	2012-2015
All	32 (31,32)	29 (28,29)	50 (50,51)	46 (46,47)	63 (62,63)	59 (58,59)
<70 years	14 (14,15)	12 (12,13)	25 (24,26)	22 (22,23)	35 (34,36)	31 (30,32)
70 to 79 years	25 (24,25)	22 (21,22)	42 (41,42)	37 (37,38)	55 (54,56)	50 (49,51)
80 to 89 years	39 (39,40)	35 (35,36)	61 (60,62)	56 (55,57)	75 (75,76)	70 (70,71)
90 years and over	58 (57,58)	53 (52,53)	80 (79,81)	76 (75,76)	91 (90,91)	88 (87,88)
Men	34 (33,34)	30 (30,31)	52 (52,53)	48 (47,49)	65 (64,66)	61 (60,61)
Women	30 (30,31)	27 (27,28)	48 (48,49)	44 (43,45)	61 (60,61)	56 (56,57)
Q1 Most affluent	29 (29,30)	27 (26,27)	47 (46,48)	43 (43,44)	59 (59,60)	56 (55,57)
Q2	31 (30,31)	28 (27,29)	49 (48,49)	45 (44,46)	61 (61,62)	57 (57,58)
Q3	32 (31,32)	29 (28,30)	50 (49,51)	46 (45,47)	63 (62,63)	59 (58,60)
Q4	33 (32,33)	30 (29,31)	51 (51,52)	48 (47,48)	64 (63,65)	60 (59,61)
Q5 Most deprived	35 (34,36)	32 (31,33)	54 (53,55)	50 (49,51)	66 (66,67)	63 (62,64)
Community diagnosis	29 (28,29)	24 (23,24)	46 (46,47)	40 (39,40)	59 (59,60)	52 (51,53)
Hospital diagnosis	39 (39,40)	34 (33,34)	60 (59,60)	53 (52,53)	72 (72,73)	66 (65,66)

Risk is reported as percentages with 95% Confidence intervals. Q; quintile

S6 Complete case analysis: Associations between population groups

Group	All Adjusted[a]	CVD Adjusted[a]	Non-CVD Adjusted[a]
**Hospital Admissions (IRR with 95% CI)**			
<70 years	1.0	1.0	1.0
70 to 79 years	1.19 (1.15, 1.24)	1.11 (1.06, 1.17)	1.23 (1.19, 1.28)
80 to 89 years	1.48 (1.42, 1.54)	1.41 (1.34, 1.50)	1.52 (1.45, 1.58)
90 years and above	1.79 (1.70, 1.90)	1.70 (1.57, 1.85)	1.83 (1.72, 1.94)
Male (ref)	1.0	1·0	1·0
Female	0·90 (0·87, 0·92)	0·86 (0·83, 0·90)	0·90 (0·88,0·93)
Socioeconomic status			
Q1 Most affluent (ref)	1·0	1·0	1·0
Q2	1.06 (1.02, 1.10)	1.08 (1.02, 1.14)	1.04 (1.00, 1.08)
Q3	1.06 (1.02, 1.10)	1.08 (1.02, 1.14)	1.05 (1.00, 1.09)
Q4	1.15 (1.10, 1.19)	1.14 (1.07, 1.21)	1.15 (1.10, 1.19)
Q5 Most deprived	1·17 (1·12,1·21)	1·16 (1·10,1·24)	1·17 (1·11,1·22)
Community (ref)	1·0	1.0	1.0
Hospital	1·54 (1·50, 1·58)	1·73 (1·66, 1·80)	1·45 (1·41, 1·49)
**Death (HR with 95% CI)**			
<70 years			
70 to 79 years	1.56 (1.50, 1.63)	1.43 (1.36, 1.51)	1.68 (1.57, 1.79)
80 to 89 years	2.55 (2.44, 2.66)	2.37 (2.24, 2.50)	2.64 (2.46, 2.83)
90 years and above	4.08 (3.87, 4.30)	3.72 (3.46, 3.99)	4.37 (3.98, 4.78)
Male (ref)	1·0	1·0	1·0
Female	0·84 (0·82, 0·87)	0·85 (0·82, 0·88)	0·84 (0·80,0·88)
Socioeconomic status			
Q1 Most affluent (ref)	1·0	1·0	1·0
Q2	1.05 (1.02, 1.09)	1.09 (1.04, 1.14)	1.01 (0.95, 1.08)
Q3	1.05 (1.02, 1.09)	1.11 (1.05, 1.16)	1.00 (0.94, 1.07)
Q4	1.11 (1.07, 1.15)	1.11 (1.06, 1.17)	1.11 (1.04, 1.18)
Q5 Most deprived	1·13 (1·09, 1·17)	1·14 (1·08, 1·20)	1·13 (1·05, 1·20)
Community (ref)	1.0	1.0	1.0
Hospital	1·51 (1·47, 1·55)	1·61 (1·56, 1·67)	1·32 (1·26, 1·37)

IRR; Incidence rate ratio, CI; Confidence interval, Q; quintile

a adjusted for age gender socioeconomic status, ethnicity, place of diagnosis, calendar year, beta-blocker, angiotensin converting enzyme inhibitor, angiotensin receptor blocker; aldosterone antagonist, aspirin, loop diuretic, number of comorbidities, ischaemic heart disease, myocardial infarction, atrial fibrillation, hypertension, diabetes, stroke, anaemia, obesity, chronic kidney disease, chronic obstructive pulmonary disease, asthma, depression, osteoarthritis, cancer and dementia, smoking, alcohol, body mass index, systolic blood pressure, cholesterol, haemoglobin and estimated glomerular filtration rate.
